# Thermal Shielding and Vapor Transport Enhancement in MOF‐Enabled Membranes for Membrane Distillation

**DOI:** 10.1002/advs.202510323

**Published:** 2025-11-16

**Authors:** Xiaolu Li, Shengming Yin, Jianhao Qian, Shahid Ali Khan, Wentao Shang, Jiawei Sun, Muhammad Usman Farid, Gang Lu, Guang Wang, Bhaskar Jyoti Deka, Jiaxin Guo, Yanguang Zhou, Sunwoo Kim, Junghwan Kim, Alicia Kyoungjin An

**Affiliations:** ^1^ School of Energy and Environment City University of Hong Kong 83 Tat Chee Avenue Kowloon Hong Kong SAR 999077 China; ^2^ State Key Laboratory of Materials Processing and Die & Mould Technology, Department of Materials Science and Engineering Huazhong University of Science and Technology Wuhan Hebei 430074 China; ^3^ Department of Civil and Environmental Engineering Rice University Houston TX 77005 USA; ^4^ Energy and Electricity Research Center International Energy College Jinan University Guangdong 519070 China; ^5^ Department of Mechanical and Aerospace Engineering The Hong Kong University of Science and Technology Clear Water Bay Kowloon Hong Kong SAR 999077 China; ^6^ Advanced Membrane Research Lab, Department of Hydrology Centre for Nanotechnology Indian Institute of Technology Roorkee Haridwar Uttarakhand 247667 India; ^7^ School of Chemical Engineering and Technology Xi’an Jiaotong University Xi’an 710049 China; ^8^ Department of Chemical and Biomolecular Engineering Yonsei University 50 Yonsei‐ro Seodaemun‐gu Seoul 03722; ^9^ Department of Chemical and Biological Engineering The Hong Kong University of Science and Technology Clear Water Bay Kowloon Hong Kong SAR 999077 China

**Keywords:** desalination, hierarchical pores, membrane distillation, thermal efficiency, ZIF‐8

## Abstract

Desalination via membrane distillation (MD) powered by low‐grade or waste heat is an emerging approach to energy‐efficient water purification. However, conventional membranes suffer from significant conductive heat loss, which limits their thermal performance. Developing membranes with low thermal conductivity is crucial for enhancing thermal efficiency. This study introduces a metal–organic framework‐enabled membrane (MEM) by embedding zeolite imidazole framework 8 (ZIF‐8) onto Poly(vinylidene fluoride‐co‐hexafluoropropylene) (PH) nanofiber. The MEM features a hierarchical porous structure, with an ultralow thermal conductivity (0.03 W m^−1^ K^−1^), thereby minimizing heat dissipation. It outperforms conventional membranes, demonstrating a vapor flux of 44.5 LMH and a thermal efficiency of 71.3%, improving MD performance. Multi‐scale simulations reveal that the dual improvements in thermal shielding and vapor flow facilitation enable the MEM to effectively harness low‐grade heat sources inaccessible to traditional membranes, positioning it as a promising solution for a sustainable water‐energy‐environment nexus.

## Graphical abstract

A metal–organic framework‐enabled membrane (MEM) with a hierarchical porous structure is developed for thermal‐efficient membrane distillation. By embedding ZIF‐8 onto PH nanofibers, the MEM achieves low thermal conductivity and significantly enhances vapor flux and thermal efficiency. Its ability to utilize low‐grade heat sources makes it a promising solution for sustainable desalination.

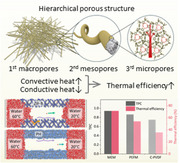

## 1 Introduction

The interdependence between water production and energy generation underscores the urgent need for advancing desalination technologies. Conventional methods, such as reverse osmosis (RO), have demonstrated efficacy in specific applications; however, they are hindered by several drawbacks, including high‐energy consumption, susceptibility to fouling, and inefficiency in treating high‐salinity brines. Consequently, there is a pressing demand for alternative technologies that can reduce energy demands and effectively manage high‐salinity water, particularly in regions reliant on seawater or brine as primary water sources. Membrane distillation (MD) has emerged as a promising solution, offering a sustainable and cost‐effective desalination approach.^[^ ^1^ ^]^ MD has a unique ability to achieve complete rejection of non‐volatile solutes, making it highly tolerant of high salt concentrations. Driven by a small temperature gradient (*ΔT*), it can integrate with waste heat or low‐grade heat (LGH) sources,^[^ ^2^, ^3^, ^4^ ^]^ thereby enhancing its cost‐effectiveness and versatility.^[^ ^5^ ^]^ However, despite these advantages, MD faces several challenges that hinder its widespread industrial adoption. In addition to durability challenges, including membrane fouling and wetting,^[^ ^4^ ^]^ insufficient thermal efficiency (*η_th_*) remains a critical bottleneck. The inherently limited transmembrane temperature difference (*ΔT*) is further diminished by substantial conductive heat losses through the membrane, thereby posing a significant impediment to process viability.^[^ ^6^, ^7^ ^]^

To address this challenge, the development of thermally insulating membranes is essential. Thermal efficiency is defined as the ratio of latent heat used for evaporation to total heat input, where the total heat encompasses both latent heat for evaporation and conductive heat losses across the membrane. Therefore, improving thermal efficiency requires enhancing the ratio of evaporation heat to conductive heat loss. Engineering membranes with ultra‐low thermal conductivity offers dual benefits.^[^ ^8^, ^9^ ^]^ First, lower thermal conductivity helps maintain a larger transmembrane temperature difference, which increases the vapor pressure difference across the membrane—the primary driving force for mass transfer—thereby resulting in higher flux. Second, lower thermal conductivity directly reduces conductive heat transfer, ensuring that more input thermal energy is utilized for evaporation rather than being lost through conduction. It is worth noting that while increasing membrane thickness can also reduce conductive heat loss, excessively thick membranes create longer diffusion pathways that impede vapor transport, consequently decreasing permeate flux and ultimately reducing thermal efficiency.^[^ ^10^ ^]^

Reducing thermal conductivity relies on manipulating intrinsic and extrinsic properties such as chemical bonding, lattice defects, nanostructure, and porosity.^[^ ^11^, ^12^, ^13^ ^]^ For instance, membranes made from polypropylene with 85% porosity exhibit half the thermal conductivity of those with 41% porosity, highlighting the direct correlation between porosity and thermal conductivity.^[^ ^14^ ^]^ In a similar vein, by altering the nanostructure and porosity, Hou et al.^[^ ^9^ ^]^ fabricated a nanocellulose membrane with remarkably low thermal conductivity and high thermal efficiency. Another zirconia ceramic membrane also achieved higher flux owing to the material's intrinsically low thermal conductivity.^[^ ^15^ ^]^ Metal–organic framework (MOF) family has also been utilized to reduce thermal conductivity.^[^ ^16^, ^17^ ^]^ For example, a zeolite imidazole framework (ZIF)‐CoZn composite membrane showed an enhanced flux due to reduced thermal conductivity.^[^ ^17^ ^]^ Additionally, a polyvinylidene fluoride (PVDF)/metal and zeolite framework‐4 (MAF‐4) composite membrane reduced its thermal conductivity to 0.04 W m^−1^ K^−1^, with a flux of 27.9 LMH.^[^ ^18^ ^]^ Another MOF‐functionalized alumina membrane demonstrated enhanced VMD performance through a two‐step molecular engineering approach, achieving reduced thermal conductivity and improved hydrophobicity with a flux of 32.3 LMH at 60 °C.^[^ ^19^ ^]^

Building on previous research, in this study, we designed and fabricated a (MOF)‐enabled membrane (MEM) by depositing ZIF‐8 nanocrystals onto electrospun poly(vinylidene fluoride‐co‐hexafluoropropylene) (PVDF‐HFP, denoted herein as PH) membranes, forming a hierarchical porous structure comprising microporous ZIF‐8, mesoporous PH fibers, and a macro‐porous interconnected fibrous network. Systematic characterization of the membrane's porous architecture and pore size distribution (PSD) demonstrated a high porosity of 90% and low thermal conductivity of 0.0316 W m^−1^ K^−1^ while maintaining a thickness of ≈100 µm and mechanical strength of 1.45 MPa. These features effectively minimize conductive heat loss and vapor transfer resistance. In MD tests, the MEM achieved a high water flux of 44.5 LMH and a thermal efficiency of 71.3%. To elucidate the underlying mechanisms, molecular dynamics and computational fluid dynamics simulations were employed to analyze mass and heat transfer, confirming the synergistic effects of the hierarchical porous structure. These findings underscore the potential of MEM to couple with low‐temperature heat sources, contributing to the water‐energy‐environment nexus by providing a sustainable desalination technique.

## 2 Results and Discussion

### 2.1 Rational Design of MEM

**Figure 1:**
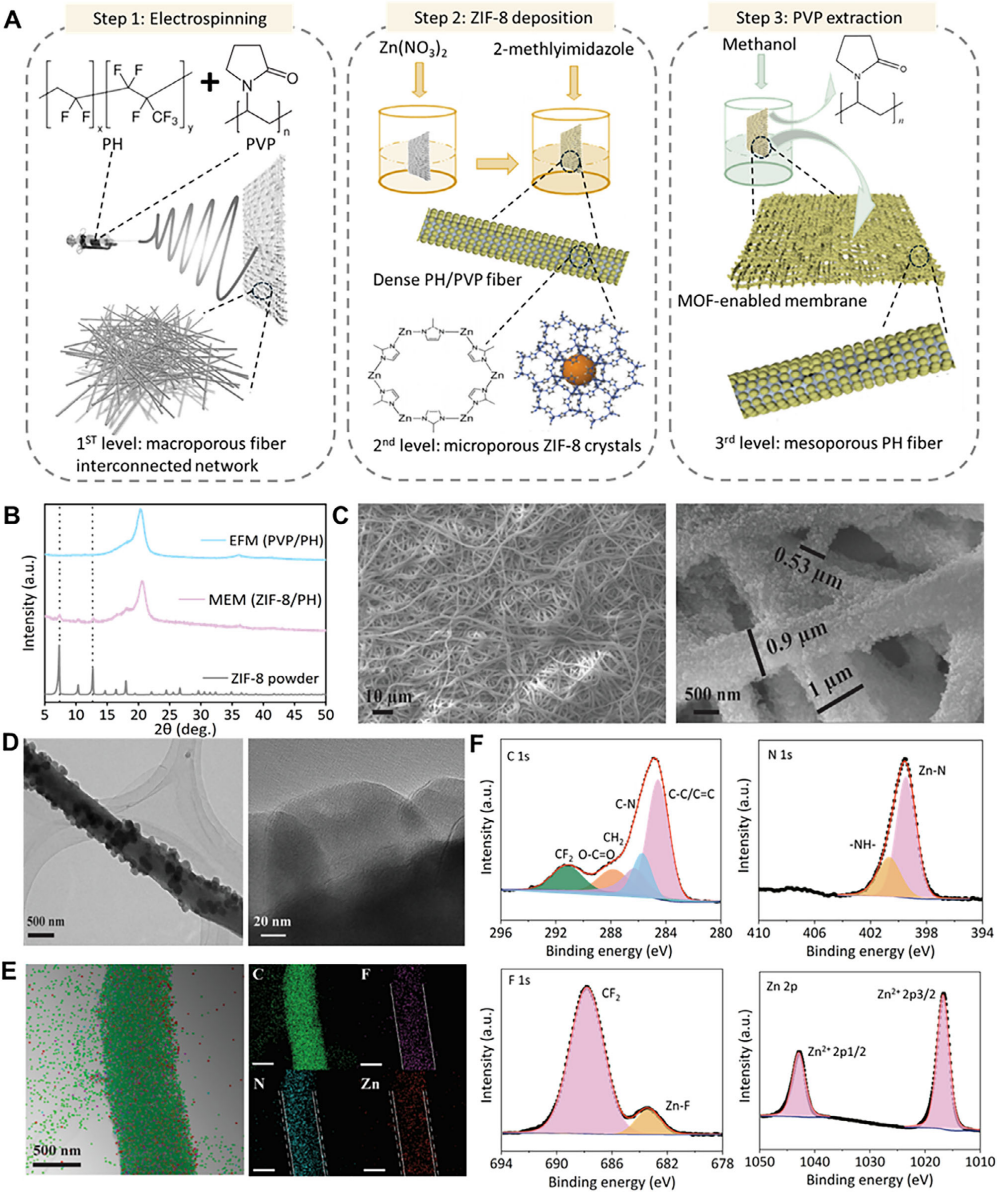
Design principles and physiochemical characterization of the MEM. A) Fabrication process of the MEM. 1st step: fabricate PH/PVP EFM by electrospinning; 2nd step: deposit microporous ZIF‐8 nanocrystals encircling PH/PVP fibers; 3rd step: create mesoporous PVDF fibers by removing PVP from PVDF/PVP blend. B) XRD patterns of ZIF‐8 powder, the EFM containing PVP and PH, and the MEM containing ZIF‐8 and PH are presented. The diffraction peaks of ZIF‐8 can be observed in the XRD pattern of the MEM. C) SEM images of the MEM show the macropores formed by overlapping nanofibers and the representative nanofiber diameters. D) TEM and HRTEM images of the MEM show ZIF‐8 nanocrystals attached to a single nanofiber. E) HAADF image with elemental mapping of the MEM, containing four main elements: C, F, N, and Zn. F) C 1s, F 1s, N 1s, and Zn 2p XPS spectra of the MEM.

The design of the MEM focuses on achieving a hierarchical porous structure that balances high thermal efficiency, water flux, and mechanical stability. The design steps are shown in **Figure** **1**A. First, the foundation of this design lies in the creation of macropores through an interconnected electrospun PH fibrous network. Leveraging the inherent hydrophobicity of PH, the electrospinning process produces an interwoven, overlapping nanofiber structure that imparts high porosity, enhanced surface roughness, reduced pore tortuosity, and superior flexibility.^[^ ^20^, ^21^, ^22^, ^23^ ^]^ To further refine the porous architecture, ZIF‐8 nanocrystals are deposited on the electrospun fibrous membrane (EFM) to introduce micropores. ZIF‐8, a standout material in the MOF family, is characterized by its large pore volume (0.762 cm^3^ g^−1^), high surface area (1799.13 m^2^ g^−1^), and exceptional chemical and thermal stability.^[^ ^24^ ^]^ Additionally, the inherent hydrophobicity of ZIF‐8, attributed to its −CH_3_‐bearing organic linker, ensures stability in aqueous environments. During membrane fabrication, ZIF‐8 nanocrystals are synthesized via chemical bath deposition at room temperature. By controlling the growth time, ZIF‑8 can be tracked from sparse coverage of the fibers after 10 min, to uniform and complete coverage after 1 h (the optimal stage, hereafter noted as MEM). After 2 h, the ZIF‐8 layer thickens and the inter‐fiber gaps become partially filled with ZIF‑8. And after 6 h of growth, overgrowth occurs, forming overly dense layers that eliminate the interconnected structure of the EFM (Figure S1). The final step of the design introduces mesopores onto the PH fibers. The fabrication of these mesoporous fibers involves first using a mixed solution of PH and Polyvinylpyrrolidone (PVP), followed by subsequent removal of PVP via methanol treatment.^[^ ^25^ ^]^ The resulting mesopores not only contribute to air storage within the membrane but also enhance surface roughness, further improving hydrophobicity.

This carefully engineered hierarchical structure endows the MEM with several advantageous properties. High porosity, High porosity, resulting from the combination of macropores, micropores, and mesopores, reduces thermal conductivity while maintaining optimal membrane thickness. The interconnected macroporous network ensures low resistance to vapor transfer, alongside good mechanical strength and flexibility. Meanwhile, the mesoporous fibers contribute to a rough surface and enhanced hydrophobicity. These synergistic properties enable the MEM to achieve both high thermal efficiency and water flux.

### 2.2 Physicochemical Characterization of MEM

Comprehensive characterizations have been conducted to analyze the structure of the MEM. The crystalline structure was studied by X‐ray diffraction (XRD). The corresponding patterns of the EFM, MEM, and ZIF‐8 powder are shown in Figure 1B. Three peaks of PH located at 18.4°, 20.6°, and 26° were observed, which correspond to (100) plane, (020) + (110) planes, and (021) plane,^[^ ^26^, ^27^ ^]^ respectively, indicating the formation of α‐crystalline phase. The α‐phase is the dominant and most stable polymorph, playing a crucial role in maintaining the structural integrity and thermal stability of the copolymer matrix. Additionally, a pattern in the 20.6 –20.8° range suggests potential β‐phase contributions embedded within the α‐phase peak at 20°. This β phase is likely induced during electrospinning by: i) electric‐field‐driven CF2 dipole/chain alignment; ii) strong mechanical stretching that promotes β‐phase formation; and iii) rapid acetone evaporation that accelerates solidification and lowers the effective crystallization temperature for β‐phase formation.^[^ ^28^ ^]^ The diffraction peaks from ZIF‐8 were seen in the MEM's pattern (7.3°, 10.4°, and 12.7°). Consistent with the XRD result, the Fourier transform infrared (FTIR) spectrum of the MEM (Figure S2, Supporting Information) appeared to have the same characteristic peaks of ZIF‐8.^[^ ^6^ ^]^ An interconnected network of randomly aligned fibers was observed in the SEM images of the EFM and MEM (Figure 1C; Figure S3, Supporting Information), indicating that the structure of the EFM is well preserved in the MEM. Fibers of the MEM had a rough surface as ZIF‐8 nanoparticles extensively cover the fibers. In contrast, the EFM's fibers were dense and had smooth surfaces. Deposition of ZIF‐8 nanoparticles also caused an increase in fiber diameter, which is illustrated in Figure 1C and Figure S3 (Supporting Information). The surface morphology of a single fiber from the MEM was observed using transmission electron microscopy (TEM) (Figure 1D). The fiber diameter was ≈0.7 µm, close to that measured from the SEM image. ZIF‐8 nanoparticles were uniformly distributed on the fiber surface, while a continuous ZIF‐8 layer had not formed. One typical ZIF‐8 nanoparticle is shown in the high‐resolution (HR) TEM image, which exhibits a polyhedron shape and a size of 80–100 nm. The small size of the particle resulted from the mild reaction conditions in the chemical bath, compared to the reaction conditions of the hydrothermal method.^[^ ^29^ ^]^ No specific lattice spacing was observed by HRTEM, corresponding to the inorganic/organic hybrid feature of MOFs.^[^ ^30^ ^]^ Elemental mapping (EM) analysis was conducted using energy‐dispersive X‐ray spectroscopy (EDX) (Figure 1E). C, F, N, and Zn were homogeneously distributed in the fiber. Zn and N were mostly present on the fiber surface, while F was found only in the core. Elemental mapping results indicated that a ZIF‐8@PH core–shell structure was obtained. This conclusion is in accordance with the fiber diameter increase found in SEM images.

The chemical composition of the MEM was also studied using X‐ray photoelectron spectroscopy (XPS). The XPS survey spectrum of the MEM presented in Figure S4 (Supporting Information) illustrates the existence of C, N, F, and Zn in the sample, which matches well with the EDX result. The chemical information of each element was analyzed by high‐resolution XPS spectra (Figure 1F). Five peaks were observed in the C 1s spectrum, which can be attributed to ZIF‐8 nanocrystals (284.5 eV for C─C/C═C, 285.7 eV for C─N in imidazole structure), the surface carbonate group (287.8 eV), and PH fibers (286.2 eV for CH_2_ and 291.1 eV for CF_2_). This result confirms the synthesis of a ZIF‐8@PH composite.^[^ ^31^, ^32^ ^]^ The F 1s spectrum includes one main peak located at 688.3 eV, which corresponds to CF_2_ in PH fibers. A small peak at 684.1 eV was also observed. A similar result was reported in a surface fluorinated TiO_2_ system, in which F was adsorbed on the TiO_2_ surface and interacted with Ti to reduce surface energy.^[^ ^33^ ^]^ Thus, we attribute this peak to the interaction between Zn and F, which may occur in the Zn(NO_3_)_2_ methanolic solution adsorption process.^[^ ^34^ ^]^

### 2.3 Characterization of Hierarchical Porous Structure

**Figure 2:**
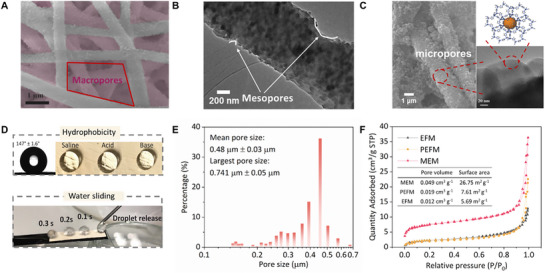
Porous structure characterization, thermal conductivity, and hydrophobicity of the MEM. A) SEM image showing macropores formed by fibrous network in the MEM. B) TEM image shows mesopores on the MEM's nanofibers. C) FE‐SEM image of ZIF‐8 assembling into a mesoporous network on the surface of PH fibres, with micropores within ZIF‐8. D) Water contact angle of the MEM indicates its hydrophobicity; Images show stable saline, acid, and base droplets on the MEM surface; An overlay image showing slippery characteristic of the MEM's surface. E) Macropore size distribution measured by liquid‐gas replacement. F) N_2_ adsorption‐desorption isotherms of MEM, PEFM, and EFM.

As discussed above, the hierarchical porous structure of a membrane is important to regulate its properties and improve its performance. The porous structure of the MEM was investigated using both visual observation and physical methods. The macropores formed by the fibrous network can be seen in the SEM image (**Figure** **2**A). The pore diameters ranged from ≈1 µm to ≈3 µm, and the stacking of the interconnected network in three dimensions would further reduce the pore size. The PSD of the MEM is in the range of 0.2–0.74 µm with its mean pore size located at 0.48 µm, which was inherited from the EFM (Figure S5, Supporting Information), within the optimized range for rapid vapor transfer. The morphology of ZIF‐8@PH fiber is presented in Figure 2B. A typical mesoporous structure is obtained due to the selective removal of PVP out of the PH/PVP composite during the methanol extraction.^[^ ^25^ ^]^ Also, evenly distributed ZIF‐8 nanocrystals had assembled into an interparticle network on the fiber surface (Figure 2C), where the topological structure of ZIF‐8 contains abundant micropores.^[^ ^35^, ^36^ ^]^ This ZIF‐8 network, on the one hand, provides abundant sites for gas adsorption to improve membrane porosity, while on the other hand, it could increase surface roughness and create air pockets to repel liquid. To understand the role of ZIF‐8 in the MEM, a PVP‐eliminated EFM (PEFM) was also fabricated as a control sample. The morphology of the PEFM was similar to that of the MEM, exhibiting mesoporous PH fibers and macroporous network, except that ZIF‐8 nanoparticles were not observed in the PEFM (Figure S6, Supporting Information).

The micro/mesoporous structure was further analyzed using N_2_ adsorption/desorption isotherms at 77 K. Both MEM and PEFM isotherm curves in Figure 2F exhibit a Type IV feature with a H3 hysteresis loop, confirming the formation of mesopores. The H3‐type hysteresis loop suggests slit‐shaped or wedge‐shaped pore geometries.^[^ ^37^ ^]^ The steep uptakes observed at low relative pressure (P/P_0_ = 0–0.01) for MEM indicates the presence of micropores, as evidenced by the significant increase in pore volume and surface area of the MEM (0.049 cm^3^ g^−1^, 26.75 m^3^ g^−1^) compared to PEFM (0.019 cm^3^ g^−1^, 7.61 m^2^ g^−1^). Conversely, the EFM exhibits a Type II isotherm, characterized by the absence of a distinct hysteresis loop and low adsorption capacity, indicating that it is primarily a macroporous material. The PSDs of the micropores and mesopores were calculated using the Horvath‐Kawazoe model and Barrett‐Joyner‐Halenda model. The mesopores’ sizes ranged from 2.17 nm to 45.44 nm. The size distribution of micropores was centered ≈1 nm, consistent with the reported aperture size of ZIF‐8.^[^ ^38^ ^]^

Subsequently, the porosity of the MEM was measured by the liquid replacement method. A porosity as high as 90% was achieved, which is close to the highest value ever reported.^[^ ^39^ ^]^ A relatively high porosity of 85% was also achieved with the PEFM, demonstrating the advantage of the electrospinning technique for fabricating membranes with high porosity. Despite such high porosity, the MEM was still flexible and stretchable with decent mechanical strength. The MEM's tensile strength, measured using a tensile strength tester, was 1.55 MPa (Figure S7, Supporting Information). Furthermore, the MEM exhibited a decent hydrophobicity with a water contact angle (WCA) of 147°. In contrast, the EFM was hydrophilic due to the existence of water‐soluble PVP (Figure S8, Supporting Information). After removing PVP, the PEFM's WCA increased to 110°, which is slightly lower than the commercial PVDF membrane (Millipore GVHP, denoted herein as C‐PVDF), as the mesoporous fibers altered the surface morphology and reduced hydrophobicity (Figure S9, Supporting Information).^[^ ^40^ ^]^ The WCA of the ZIF‐8 powder pellet indicated its hydrophobic nature, owing to the hydrophobic compositions of conjugated imidazolate rings.^[^ ^41^, ^42^ ^]^ Therefore, we can attribute the decent hydrophobicity of the MEM to the mesoporous network of ZIF‐8 nanocrystals assembled on the fiber surface. The exposed edges of ZIF‐8 nanocrystals repel water, rather than allowing water to spread on its surface. The enhanced roughness from ZIF‐8 nanocrystal assembly also contributed to the improved hydrophobicity by creating air pockets.^[^ ^43^, ^44^ ^]^ ZIF‐8′s chemical stability in different solvents allows saline, acidic, and basic solutions to form stable droplets on the MEM's surface for more than 30 min (Figure 2D), suggesting the MEM's potential for application in acidic/basic wastewater treatments. Besides the static WCA test, the MEM's slippery surface also supported its hydrophobicity. As can be seen in Figure 2D, water droplets quickly slip over the membrane surface after release. Other characteristics of the membranes utilized or mentioned in this study are summarized in Table S2 (Supporting Information).

### 2.4 High Flux and Thermal Efficiency of MEM

**Figure 3:**
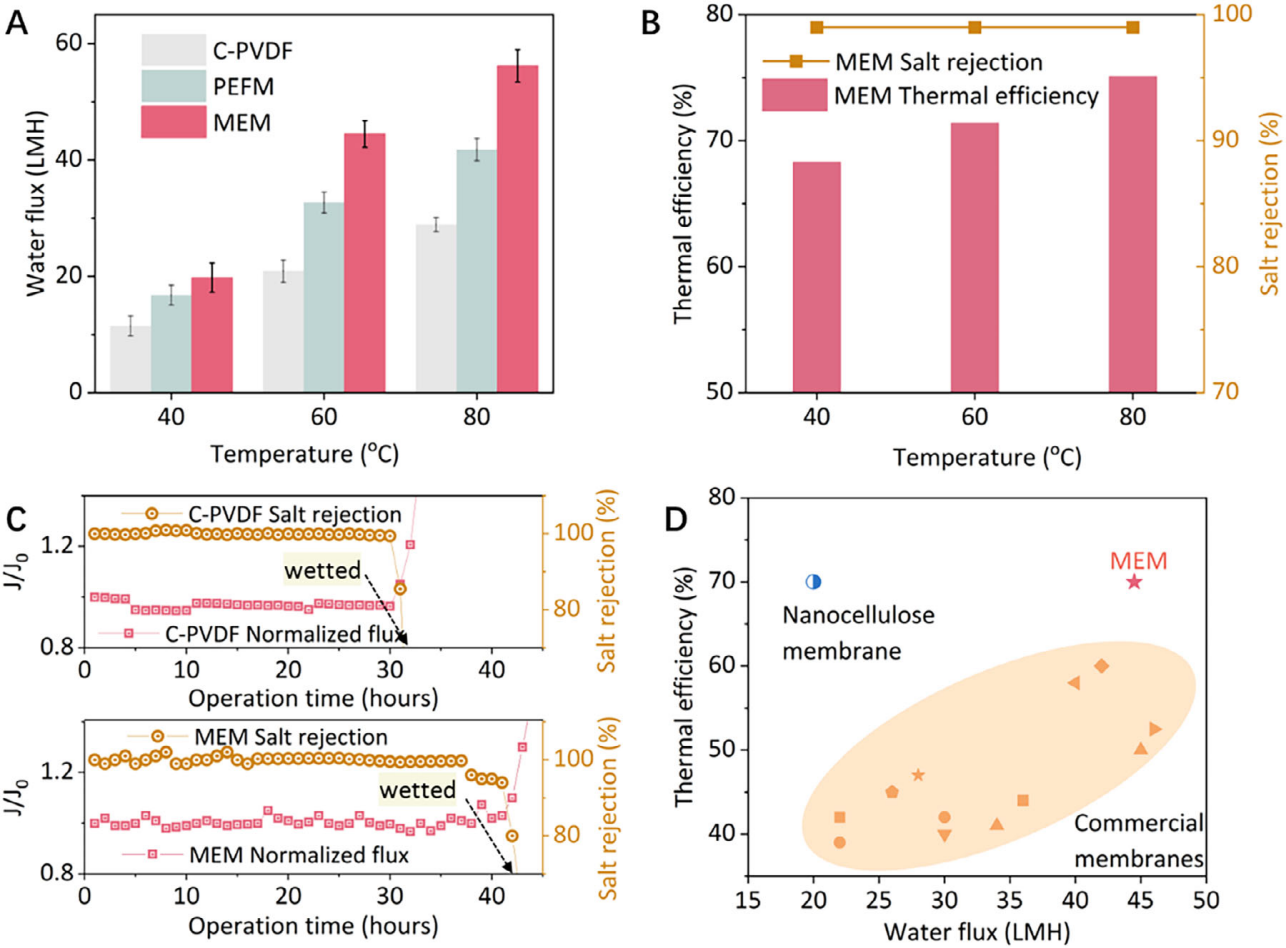
MD performance of the MEM compared with other membranes. A) Water flux for the MEM, PEFM, and C‐PVDF‐0.45µm at feed temperatures of 40, 60, and 80 °C. B) Salt rejection, and thermal efficiency for the MEM at feed temperatures of 40, 60, and 80 °C. C) Long‐term durability test of the MEM and C‐PVDF. D) Thermal efficiency and water flux of the MEM and other reported membranes in literature.^[^ ^14^ ^]^

The MEM was utilized for MD operation with simulated seawater in a customized direct‐contact MD (DCMD) setup. The membrane module was equipped with eight thermocouple sensors for monitoring of temperature variations online. Four sensors were positioned at the two inlets and two outlets, while the remaining four were attached at the membrane surface (Figure S10, Supporting Information).^[^ ^45^, ^46^, ^47^ ^]^As shown in **Figure** **3**A, the MEM showed higher water flux than both the PEFM and C‐PVDF. The MEM's salt rejection remained constant at 99% for all operation conditions, indicating its good desalination capability. MEM achieves comparable flux at 40 °C to that of C‐PVDF at 60 °C, indicating suitability for LGH utilization where operating temperatures are constrained. The thermal efficiency of the MEM increased with higher feed temperatures, and the highest thermal efficiency of 75.1% was achieved when the feed was 80 °C (Figure 3B). The dependence of thermal efficiency on feed temperature is consistent with previous findings.^[^ ^10^ ^]^ The increase in thermal efficiency with feed temperature is due to the exponential rise in vapor pressure difference, which drives higher vapor flux, while conductive heat loss increases only linearly (Equation 5). Besides superior flux and thermal efficiency, the MEM also showed good durability.

A long‐term durability test was conducted for both C‐PVDF and MEM, respectively (Figure 3C). After roughly 30 h, C‐PVDF experienced a flux increase accompanied by a salt rejection drop, indicating membrane wetting had occurred, meanwhile MEM maintained unchanged flux and high rejection. Post‐operation SEM image in Figure S11 (Supporting Information) confirms that the ZIF‐8 coating remains intact after MD desalination testing, suggesting that mesoporous embedding effectively stabilizes the ZIF‐8 particles during operation. MEM sustained operation for 10 h longer than C‑PVDF. A slight decline in flux was observed during the final 4 h, which may be attributed to salt crystallization within the micropores. Accordingly, the potential wetting mechanism is proposed as follows: under combined thermal–salinity stress, ZIF‑8 undergoes partial degradation and/or microcracking, allowing saline solution to infiltrate. This process initially occurs within the ZIF‑8 domains and is exacerbated to the ZIF‑8/PH interface, which further deforms the micro/mesopores. As a result, the membrane's hydrophobicity gradually declines, ultimately failing to prevent liquid water penetration.^[^ ^48^ ^]^ Despite this, MEM maintains comparable stability over previously reported MOF‑functionalized membranes and several PVDF‑based membranes (Table S3, Supporting Information). Future studies should focus on two aspects: reinforcing long‐term robustness while maintaining high flux, and developing an effective membrane cleaning approach, which remain absent in MD unlike other commercialized membrane processes. If cleaning can be implemented before wetting occurs, the performance advantage of MEM over C‐PVDF can be more pronounced.

Compared to commercial membranes, the flux and thermal efficiency of the MEM are highly competitive. In Figure 3D, the commercial membranes within the orange area have high thermal conductivity, which reduces thermal efficiency in two ways: first, it worsens temperature polarization thus reducing evaporative heat transfer; second, it increases heat conduction across the membrane. Another scenario is represented by the membrane in the upper left corner, where thermal conductivity is reduced at the expense of excessive membrane thickness.^[^ ^9^ ^]^ For such thick membranes, both evaporative heat transfer and heat loss are diminished. Consequently, although thermal efficiency appears high, the flux is substantially lower than that of typical commercial membranes. In contrast, the MEM positioned in the upper right corner maintains high flux while achieving high thermal efficiency, overcoming traditional performance trade‐offs.

**Figure 4:**
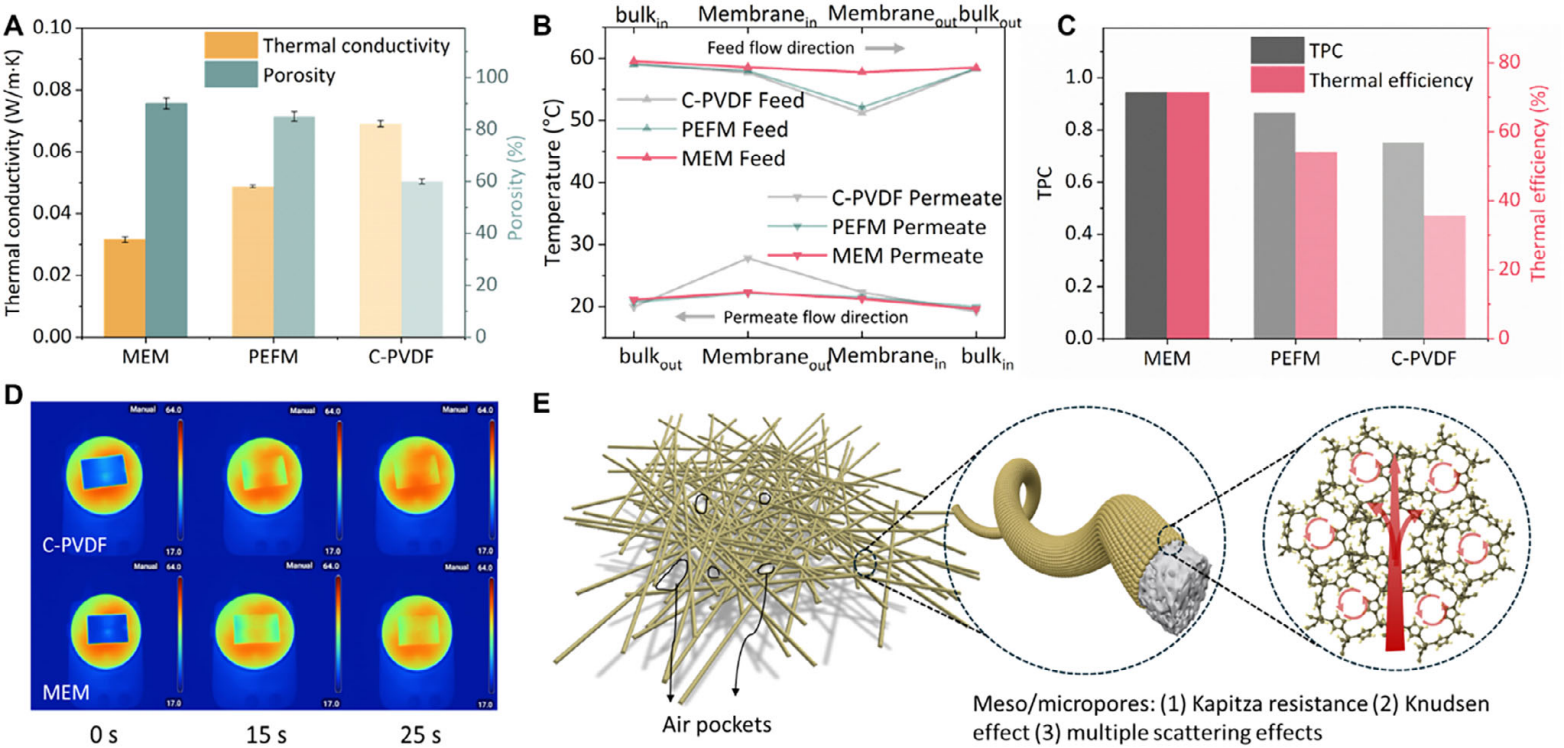
Low conductivity of the MEM. A) Thermal conductivity and porosity of the MEM, PEFM, and C‐PVDF. B) Temperature profiles of three membranes during MD operation. C) Temperature polarization coefficients (TPC) and thermal efficiencies of the MEM, PEFM, and C‐PVDF when feed and permeate were at 60 and 20 °C. D) Infrared images of the MEM and C‐PVDF. E) Proposed thermal shielding mechanism of the MEM.

The superior overall performance of MEM can be attributed to its low thermal conductivity while maintaining a relatively thin thickness. To validate, a thermal conductivity analyzer was utilized to measure the thermal conductivity of the MEM, PEFM, and C‐PVDF membranes (**Figure** **4**A; Figure S12, Supporting Information). Compared to the C‐PVDF, the MEM exhibited a reduction of over 50% in thermal conductivity, with a value of only 0.0316 W m^−1^ K^−1^. The MEM also demonstrated the highest porosity, reaching 90%. The enhanced porosity of MEM compared to PEFM results from the abundant micropores introduced by the ZIF‐8 coating. Importantly, the ZIF‐8 layer forms on the membrane surface and within the structure without blocking the existing macropores and mesopores, thereby maximizing the overall porosity. During DCMD operation, this difference in thermal conductivity is reflected in the temperature profiles recorded by the thermocouples (Figure 4B). While all membranes maintain similar feed temperatures (≈60 °C), MEM exhibits the largest temperature difference across the membrane, followed by PEFM, then C‐PVDF. This greater temperature difference corresponds to a larger driving force for mass transfer, a higher temperature polarization coefficient (TPC), and a higher thermal efficiency (see Figure 4C). While conductive heat loss increases in absolute terms with higher temperature differences, it does so at a slower rate compared to convective heat, resulting in improved thermal efficiency. The infrared (IR) photographs in Figure 4D are direct visual evidence demonstrating the better thermal insulation capacity of the MEM. When both membranes were subjected to the same heat source, the surface temperature increase of the MEM was lower than that of the C‐PVDF, which indicates a lower thermal conductivity of the MEM.

The hierarchical porous structure of the MEM contributes to its low thermal conductivity, making it an effective thermal insulator (Figure 4E). Similar heat transfer mechanisms for such structures have been discussed in previous studies.^[^ ^49^, ^50^ ^]^ The first‐tier macropores within the interconnected fibrous network provide fundamental thermal resistance by creating abundant air gaps with inherently low thermal conductivity. Phonon conduction through the solid framework is interrupted by gas‐filled pores, where thermal resistance at the solid‐gas interfaces arises from the mismatch between the solid skeleton (phonon conduction) and air (molecular collisions).^[^ ^51^, ^52^ ^]^ The second‐tier mesopores on fiber surfaces provide additional air storage spaces and enhanced surface roughness, which reduces effective interfacial contact area for heat transfer. Mesopores also facilitate multiple scattering effects, with phonon scattering at rough surfaces, extending heat conduction pathways and creating tortuous thermal routes.^[^ ^53^ ^]^ The third‐tier micropores within ZIF‐8 offer another thermal resistance unit at the molecular level. At nanoscale pores, the Knudsen effect becomes dominant when pore diameter approaches the mean free path of gas molecules, resulting in molecule‐wall collisions rather than intermolecular collisions and reducing the gas‐phase thermal conductivity. Simultaneously, interfacial thermal resistance (Kapitza resistance) at ZIF‐8/PH interface further impedes phonon transmission.^[^ ^54^, ^55^, ^56^ ^]^ These synergistic effects allow the MEM to exhibit enhanced convective heat transfer (i.e., increased flux) as temperature rises, while wall‐molecular collisions in micropores inhibit gas‐phase conductive heat transfer. In contrast, commercial membranes with only sparse macropores lack channels and sufficient resistance to conduction. Consequently, the MEM demonstrates superior thermal management compared to commercial membranes when coupled with LGH operation.

### 2.5 Mechanistic Insight using Multi‐Scale Simulations

**Figure 5:**
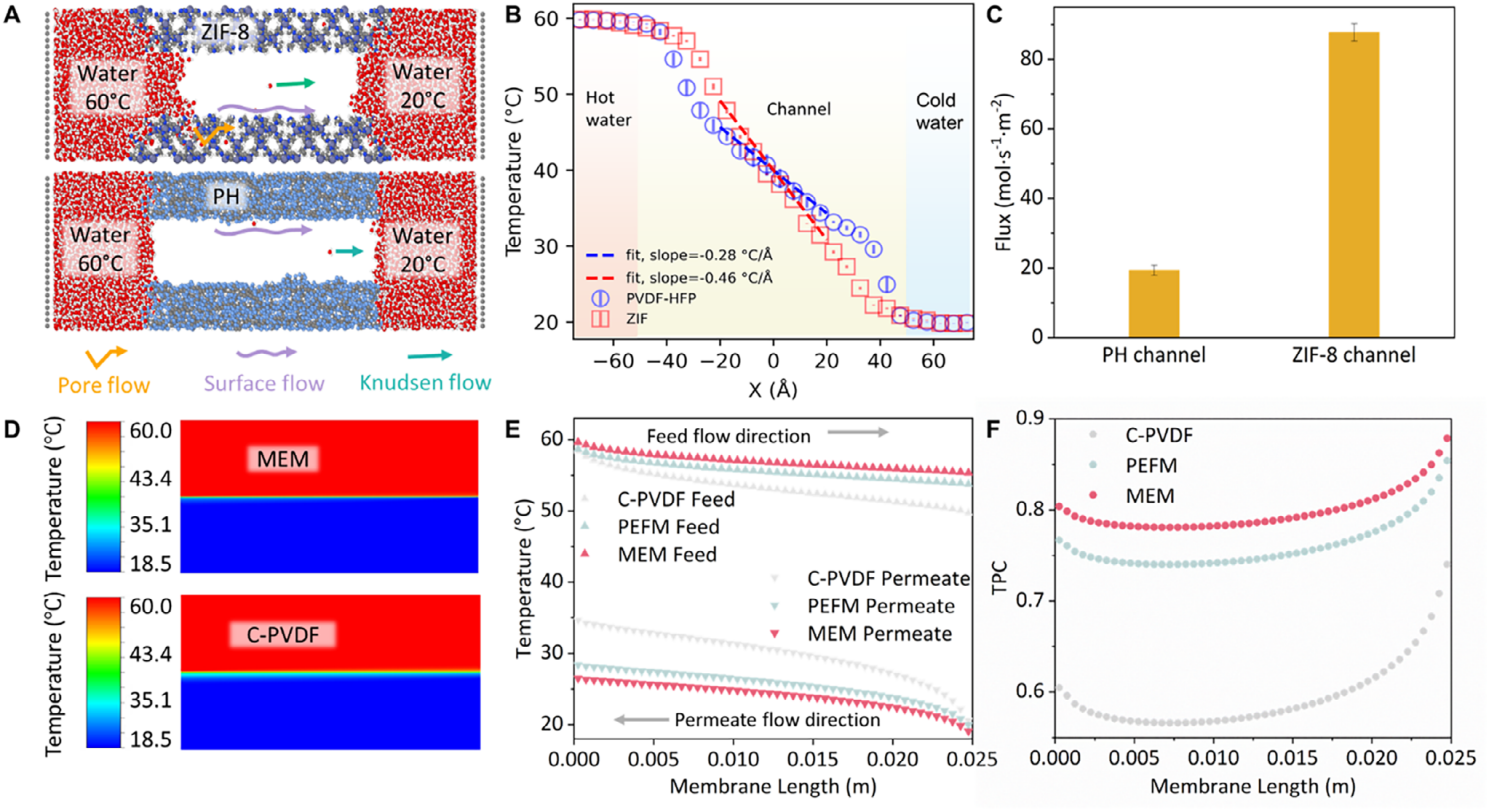
Molecular dynamics and CFD simulations. A) Molecular dynamics model of different water transport mechanisms within ZIF‐8 and PH nanoscale channels. B) Molecular dynamics simulation of wall temperature profiles of PH and ZIF‐8. C) Molecular dynamics simulation of water flux in PH channels and ZIF‐8 channels. D) CFD‐simulated temperature contours of the MEM and C‐PVDF during DCMD. E) CFD‐simulated temperature profiles over the membrane surface of the C‐PVDF, PEFM, and MEM. F) CFD‐simulated TPC on the surfaces of the C‐PVDF, PEFM, and MEM.

Molecular dynamics simulations (**Figure** **5**A; Figure S13, Supporting Information) were performed to evaluate intrinsic thermal conductivity of PH and ZIF‐8. Figure 5B shows the temperature distribution profiles from ≈60 to 20 °C for PH and ZIF‐8 materials. The data in the region from x = −20 to 20 Å, which is well separated from the water–membrane interfaces located ≈−50 and 50 Å, were fitted. A noticeable slope difference was obtained, with PH ‐0.28 °CÅ^−1^ and ZIF‐8 ‐0.46 °CÅ^−1^. Under identical heat flux, the steeper temperature gradient in ZIF‐8 indicates its better thermal insulating capability compared to PH. The difference in thermal conductivity may be attributed to different heat conduction mechanisms: in PH, heat conduction occurs primarily through molecular vibrations along the polymer chains,^[^ ^57^ ^]^ creating continuous conduction pathways; in contrast, ZIF‐8′s porous structure interrupts heat conduction, with phonons scattering at the pore‐wall interfaces, thus reducing thermal conductivity.^[^ ^58^ ^]^

Molecular dynamics further indicated that the ZIF‐8 nanocrystal structure may provide an additional pathway for water molecule transfer, which contributes to the MEM's higher flux. Simulation results revealed that the number of water vapor molecules transported through the ZIF‐8 nanochannels is notably higher compared to the PH channels (Figure 5C). Enhanced water vapor permeability through ZIF‐8 channels has been observed and discussed in previous studies on ZIF‐8‐based composite membranes.^[^ ^59^, ^60^, ^61^, ^62^, ^63^ ^]^ In addition to surface flow and Knudsen flow, which are also present in PH channels, ZIF‐8 channels exhibit an additional pore flow, which refers to the transport of water vapor through the topological structure of ZIF‐8. ZIF‐8 features a topology with large pores (11.6 Å) connected by smaller pores (3.4 Å). This molecular sieve effect enables water molecules, with a kinetic diameter of 2.8 Å, to slide through the framework.^[^ ^24^ ^]^ Additionally, the hydrophobic pore walls prevent water molecules from adhering to the surface, thereby enabling them to transport within the channels.^[^ ^60^, ^62^ ^]^

Figure 5D illustrates the temperature contours for the hot and cold channels of the MEM and C‐PVDF under same DCMD operation, derived by solving the Navier‐Stokes, heat, and mass transfer equations. The temperature transition zone on both sides of the MEM is thinner compared to that of C‐PVDF. The temperature variation in the feed and permeate layers is steeper. The underlying mechanism is that MEM is more effective in shielding heat, leading to a larger temperature gradient in the DCMD process. As a result, among the three membranes analyzed (Figure S14, Supporting Information), the MEM exhibited the highest overall efficiency. The CFD model accuracy was validated against experimental data (Figure S15, Supporting Information). Further details of the transmembrane temperature distribution are given in Figure 5E. The superior thermal insulation of the MEM effectively impeded heat conduction, preserving the transmembrane temperature difference. This is reflected in the largest gap between the smoother feed and permeate curves. TPC at each point on the membrane was also simulated. The TPCs of all three membranes followed a distinct U‐shaped curve, which is likely due to insufficient mass and heat transfer at the inlet where the fluid first contacts the membrane, resulting in a relatively large transmembrane temperature difference and higher TPC.

## 3 Conclusion

In this work, we designed and fabricated a novel MEM with a hierarchical porous structure for DCMD. The introduction of ZIF‐8 enhanced the MEM's porosity and reduced its thermal conductivity, while the mesoporous ZIF‐8 network, assembled on the PH fibers, enhanced surface roughness and boosted hydrophobicity. The formation of large macropores and an interconnected pore network facilitated efficient vapor transfer by reducing resistance. Its controlled thickness and decent mechanical strength further contributed to its overall performance. With a water flux of 44.5 LMH and a thermal efficiency of 71.3%, our MEM demonstrated the potential to achieve considerable flux at lower operating temperatures, making it well‐suited for integration with low‐grade heat sources. Molecular dynamics simulations revealed the role of ZIF‐8 layer in providing additional pathways for water molecule transport. Computational fluid dynamics (CFD) simulations bridged the molecular‐scale insights with experiments by visualizing the temperature distributions, further reinforcing the reliability of the MEM's performance. This study is significant as it expands the application of novel nanomaterials with commercial potential by providing an energy‐efficient and scalable solution for sustainable saline water treatment, with the ultimate goal to advance the synergy across the water‐energy‐environment nexus and address global challenges in saline water management. Future research will focus on investigating the MEM's long‐term operational stability, assessing its performance under hypersaline conditions, and further enhancing its anti‐fouling and anti‐wetting properties.

## 4 Experimental Section

### PH/PVP EFM Fabrication

The PH/PVP EFM was fabricated using the electrospinning process (Figure 1A). Typically, 1.5 g PVDF‐HFP (Mw = 455000 gmol^−1^, Sigma–Aldrich, 99%) and 0.15 g PVP (Mw = 58000 gmol^−1^, Aldrich, 99%) were mixed in 8.5 g N,N‐dimethylformamide (DMF)/acetone mixture (mass ratio: 7:3, analytical grade, ACROS) at 60 °C with vigorous stirring to form a 15 wt.% homogeneous polymer solution. Then, the solution was transferred into a syringe with a stainless steel needle with a 22 Gauge tip, which was linked to a high‐voltage power supply. The electrospinning process was conducted under 50% humidity in an ET‐2535 electrospinner (Beijing Ucalery Technology) at ambient conditions. The voltage difference is 23 kV, while maintaining a tip‐to‐collector distance of 15 cm. The injection rate of the mixed polymer was kept at 1.4 mLh^−1^, and the drawn polymer fibers were collected using an Al foil‐covered rotating drum at 120 rpm. After a 6 h electrospinning process, the white PH /PVP EFM was taken out and dried in a 60 °C oven overnight to remove residual solvent.

### ZIF‐8@PH MEM Fabrication

ZIF‐8 nanocrystals were deposited on the PH/PVP EFM in a solution process at ambient conditions. The as‐prepared EFM was immersed in a 0.1 M Zn(NO_3_)_2_·6H_2_O (Zinc nitrate hexahydrate, Mw = 297.4 gmol^−1^, Sigma–Aldrich, 98%) methanolic (analytical grade, ACROS) solution for 10 min. Then, the EFM was taken out, air‐dried for a few minutes and immersed in a 0.1 M 2‐methylimidazole (Mw = 82.10 gmol^−1^, Aldrich, 99%) methanol solution for another 10 min. Then, the EFM was transferred to the 2‐methylimidazole solution, and the Zn(NO_3_)_2_ solution was subsequently added and gently shaken until the mixed solution turned turbid. Then the mixture was allowed to stand for a certain time for the growth of ZIF‐8 onto the EFM's nanofibers. Subsequently, the as‐prepared ZIF‐8/EFM was immersed in methanol solution and kept in a 60 °C oven. After 12 h, the methanol was replaced by a fresh one and heated for another 12 h. The purpose of this process is to dissolve PVP from the PH/PVP blend, leaving pores in the fibers. The as‐prepared MEM was further rinsed with methanol several times to remove any unreacted species and dried in air at 60 °C for at least 12 h. Finally, the MEM was activated in vacuum at 100 °C for 48 h. After activation, the yellow MEM became hydrophobic as the guest molecule, methanol, was removed from the pores of ZIF‐8 structure. Separately, a PEFM constructed by interconnected mesoporous PH fibers was fabricated and used as a control sample. The fabrication process is similar to that for the MEM, except that ZIF‐8 nanocrystals are not deposited on the EFM. After PVP was extracted by methanol, the PEFM was heated in vacuum at 100 °C for 48 h to eliminate methanol in the mesopores.

### Physicochemical Characterization of Membranes

The chemical structure of the prepared membranes was characterized by utilizing an X‐ray diffractometer (XRD). The surface structure and morphologies of MEM (including surface and cross‐section) were observed by a FESEM (JEOL JSM‐7800F). TEM images with HRTEM mode were obtained on JEM‐2100F field emission electron microscopy. High‐angle annular dark‐field (HAADF) scanning images and EDS analysis were obtained at the same TEM facility. XPS analysis was conducted using a monochromated Al Kα X‐ray source to analyze the elemental composition of the membrane surface. The tensile strength was determined by a mechanical strength testing instrument (Lloyd LS1, U.S.). The PSD of the MEM's macropores, which are inherited from the EFM substrate, was measured by gas–liquid replacement method using a porometer from GmbH, Germany. Porefil from the same supplier was used as a wetting agent. The same porometer was also utilized to measure the liquid entry pressure (LEP). The porosity (ε) was measured by the gravimetric method. A membrane sample (3 cm × 3 cm) was completely dried and subsequently weighed (m_dry_). Then, it was immersed in 2‐propanol (99%, ACROS) until fully wet (at least one day). After infiltration, the residue liquid on the surface was removed and then weighed again (m_wet_). In the end, the porosity was obtained using the following equation:

(1) $$\varepsilon =\frac{m_{wet}-m_{dry}}{\rho_{IPA}}/\left[\frac{m_{wet}-m_{dry}}{\rho_{IPA}}+\frac{m_{dry}}{\rho_{mem}}\right]$$

where ρ_IPA_ and ρ_mem_ represent the density (g cm^−3^) of 2‐propanol and membrane material, respectively. The contact angle was measured using an EasyDrop instrument (Kruss, Germany), which captured the droplet's contact with a surface via a video digitizer for subsequent analysis, and determined through the sessile drop technique. Brunauer–Emmet–Teller (BET) test was employed to measure the nitrogen adsorption/ desorption isotherm at 77K. The thermal conductivity of membranes was measured based on the transient plane source (TPS) method^[^ ^64^ ^]^ by a thermal conductivity analyzer (DRE‐III, Xiangyi instrument). All measurements were conducted in ambient air at room temperature and atmospheric pressure with dry membrane samples. The membrane with a thickness of ≈100 um was cut into different pieces in a size of 1 × 1 cm^2^, and steel was used as the reference sample. A constant power (0.1W) was applied to the heater to generate a temperature rise of ≈5K. All values reported for these measurements are the average values of three independent measurements. In addition, infrared thermographs (T630sc, FLIR) were captured when the sample was adhered to a glass slide and placed on the same hot plate for heating. Thermal gravimetric analysis (TGA) was conducted under air condition, with temperatures rising from 20 to 800 °C and a constant flow rate of 100 mL min^−1^.

### MEM Performance Test

DCMD tests were conducted to test the applicability and performance of the MEM for distillation. A 2.5 cm × 1.5 cm customized acrylic module was designed and used for all MD tests. During operation, two sets of thermocouple sensors are deployed in the module to monitor temperature changes in real‐time. Simulated seawater containing 35 g L^−1^ NaCl was heated up using a hot plate, while the deionized (DI) water was used as permeate and was kept at 20 °C by a chiller. Feed and permeate liquid were pumped to circulate across the membrane at a velocity of 0.4 L min^−1^. The water flux, *J*, (kg m^−2^ h^−1^, or LMH) was calculated by Equation (2), where the weight difference on the permeate side was recorded by a balance with the data logging function.

(2) $$J=\frac{\Delta m}{A\times t}$$

where *Δm*, *A*, and *t* represent the increase in permeate mass, effective membrane area and time interval, respectively. Salt rejection *R* (%) was obtained by Equation (3):

(3) $$R\left(\%\right)=\left(1-\frac{V_{2}Y_{2}-V_{1}Y_{1}}{\left(V_{2}-V_{1}\right)Y_{f}}\right)\times 100\%$$

where *V_1_* and *Y_1_* represent the volume and electrical conductivity of permeate solution at time 1, respectively; *V_2_* and *Y_2_* are the volume and electrical conductivity of permeate solution at time 2, respectively; and *Y_f_* is the electrical conductivity of the initial feed solution. In addition, a long‐term stability test was conducted under DCMD operation conditions until the membrane became wet and the salt rejection dropped dramatically. Millipore GVHP (PVDF membrane with 0.45 µm pore size) was selected as the commercial membrane benchmark as it has been widely studied in previous research.

### Temperature Polarization Coefficient and Thermal Efficiency Calculation

The temperature polarization coefficient (TPC) in DCMD can be calculated by Equation (4):

(4) $$TPC=\frac{T_{m,f}-T_{m,p}}{T_{b,f}-T_{b,p}}$$

where *T_m,f_* and *T_m,p_* are the temperature of membrane surface in the feed side and permeate side, respectively, and *T_b,f_* and *T_b,p_* are the temperature of feed bulk solution and permeate bulk solution, respectively.^[^ ^65^, ^66^ ^]^ During the experiment, *T_m,f_* and *T_m,p_* were recorded using thermocouples, and *T_b,f_* and *T_b,p_* were maintained constant using a hot plate and a chiller.

Thermal efficiency (η) was used to quantify heat loss using Equation (5):

(5) $$\eta_{\mathrm{th}}=\frac{J\Delta H_{\nu }}{J\Delta H_{v}+\frac{k_{m}}{\delta }\left(T_{m,f}-T_{m,p}\right)}\;$$

where *J* is water flux (LMH), *∆H_v_* is latent heat of water vapor (kJ kg^−1^), *k_m_* is the conductivity of membrane (W m^−1^ K^−1^), and *δ* is the membrane thickness (m).^[^ ^67^ ^]^

### Discovering Mass and Heat Transfer Mechanisms using Multi‐Scale Simulations

We employed molecular dynamics and 2D CFD simulations to investigate the mass and heat transfer mechanisms of the MEM at both microscopic and macroscopic scales. First, molecular dynamics simulations were used to investigate water transport mechanisms, flux, and temperature profiles within nanoscale channels constructed with ZIF‐8 or PH walls. ZIF‐8 channels were created by extending the structure along three axes, while PH walls consisted of polymer chains annealed into stable configurations.^[^ ^68^ ^]^ A 2 nm‐high vacuum layer defined the nanoscale channels, and water layers were placed on both sides under 1 bar pressure with temperatures set at 60 °C (hot side) and 20 °C (cold side). After a 10 ns relaxation, simulations ran for 60 ns to monitor water transport and temperature profiles, and membrane flux was determined by tracking water molecule movement. Simulations were performed using a large‐scale atomic/molecular massively parallel simulator (LAMMPS),^[^ ^69^ ^]^ with a Berendsen thermostat regulating temperatures,^[^ ^70^ ^]^ and force fields sourced from literature for ZIF‐8,^[^ ^24^, ^71^ ^]^ OPLS‐AA for PH,^[^ ^72^, ^73^ ^]^ and SPC/E for water.^[^ ^74^ ^]^

Subsequently, a 2D‐CFD model was utilized to simulate real testing scenarios, integrating the dynamics of heat and mass transfer, including convection, conduction, and evaporative heat loss, and capturing molecular and Knudsen diffusion within the membrane pores. This model enables a comprehensive understanding of the fluid flow behavior, temperature distribution, and concentration polarization effects that occur during the DCMD process. Detailed simulation methods can be found in Supporting Information.

## Conflict of Interest

The authors declare no conflict of interest.

## Author Contributions

X.L., S.Y., and J.Q. contributed equally to this work. X.L. and S.Y. designed and fabricated the membrane, finished experiments and data analysis, and drafted the manuscript. A.K.A. supervised the study by securing funding, managing the project, conceptualizing the study, developing the methodology, and reviewing and editing the manuscript. M.U.F. refined the concept and contributed to writing, reviewing, and editing. J.Q. conducted the molecular dynamics simulations, while S.A.K. performed the CFD simulations.G.W. and Y.Z. contributed thermal conductivity measurements and data analysis. W.S., J.S., G.L., B.J.D., J.G., S.K., and J.K assisted with characterization analysis or manuscript preparation and revision. All coauthors analysed and interpreted the results and provided comments on the manuscript.

## Supporting information



Supporting Information

## Data Availability

All data needed to evaluate the conclusions in the paper are present in the paper and/or the Supporting Information.

## References

[1] P. J. J. Alvarez , C. K. Chan , M. Elimelech , N. J. Halas , D. Villagrán , Nat. Nanotechnol. 2018, 13, 634. 30082804 10.1038/s41565-018-0203-2

[2] A. Deshmukh , C. Boo , V. Karanikola , S. Lin , A. P. Straub , T. Tong , D. M. Warsinger , M. Elimelech , Energy Environ. Sci. 2018, 11, 1177.

[3] A. Hussain , A. Janson , J. M. Matar , S. Adham , Emergent Mater 2022, 5, 347.

[4] M. Qasim , I. U. Samad , N. A. Darwish , N. Hilal , Desalination 2021, 518, 115168.

[5] S. Chu , A. Majumdar , Nature 2012, 488, 294. 22895334 10.1038/nature11475

[6] F. Yang , H. Mu , C. Wang , L. Xiang , K.e X. Yao , L. Liu , Y. Yang , Y.u Han , Y. Li , Y. Pan , Chem. Mater. 2018, 30, 3467.

[7] S. Al‐Obaidani , E. Curcio , F. Macedonio , G. Di Profio , H. Al‐Hinai , E. Drioli , J. Memb. Sci. 2008, 323, 85.

[8] Z. Li , Y. Peng , Y. Dong , H. Fan , P. Chen , L. Qiu , Q. Jiang , Appl. Surf. Sci. 2014, 317, 338.

[9] D. Hou , T. Li , X. Chen , S. He , J. Dai , S. A. Mofid , D. Hou , A. Iddya , D. Jassby , R. Yang , L. Hu , Z. J. Ren , Sci. Adv. 2019, 5, aaw3203. 10.1126/sciadv.aaw3203 PMC6677554 31414047

[10] J. Zuo , S. Bonyadi , T. S. Chung , J. Memb. Sci. 2016, 497, 239.

[11] H.‐B. Zhao , M. Chen , H.‐B. Chen , ACS Sustain. Chem. Eng 2017, 5, 7012.

[12] T. Li , J. Song , X. Zhao , Z. Yang , G. Pastel , S. Xu , C. Jia , J. Dai , C. Chen , A. Gong , F. Jiang , Sci. Adv. 2018, 4, eaar3724, 10.1126/sciadv.aar3724. 29536048 PMC5844708

[13] H.‐J. Zhan , K.‐J. Wu , Y.a‐L. Hu , J.‐W. Liu , H. Li , X.u Guo , J. Xu , Y. Yang , Z.‐L. Yu , H.‐L. Gao , X.i‐S. Luo , J.‐F.u Chen , Y. Ni , S.‐H. Yu , Chem 2019, 5, 1871.

[14] J. Vanneste , J. A. Bush , K. L. Hickenbottom , C. A. Marks , D. Jassby , C. S. Turchi , T. Y. Cath , J Memb Sci 2018, 548, 298.

[15] M. W. Boey , S. A. Khan , X. Li , J. Sun , M. U. Farid , A. K. An , Chem. Eng. J. 2025, 512, 162582.

[16] D. Cheng , L. Zhao , N. Li , S. J. D. Smith , D. Wu , J. Zhang , D. Ng , C. Wu , M. R. Martinez , M. P. Batten , Z. Xie , J Memb Sci 2019, 588, 117204.

[17] S. Jiang , S. Liang , C. Hu , Y. Fan , Z. Su , Z. Geng , C. Wang , Sep. Purif. Technol. 2024, 344, 127280.

[18] R. Wu , Y. Tan , F. Meng , Y. Zhang , Y. X. Huang , Desalination 2022, 540, 116013.

[19] J. Zuo , T.‐S. Chung , Water (Basel) 2016, 8, 586.

[20] J. Xue , J. Xie , W. Liu , Y. Xia , Acc. Chem. Res. 2017, 50, 1976. 28777535 10.1021/acs.accounts.7b00218 PMC6589094

[21] L.‐L. Min , Z.‐H. Yuan , L.‐B. Zhong , Q. Liu , R.‐X. Wu , Y.‐M. Zheng , Chem. Eng. J. 2015, 267, 132.

[22] N. Radacsi , F. D. Campos , C. R. I. Chisholm , K. P. Giapis , Nat. Commun. 2018, 9, 4740. 30413717 10.1038/s41467-018-07243-5 PMC6226441

[23] J. Guo , M. Jiang , X. Li , M. U. Farid , B. J. Deka , B. Zhang , J. Sun , Z. Wang , C. Yi , P. W. Wong , S. Jeong , B. Gu , A. K. An , Nat. Commun. 2024, 15, 7750. 39237575 10.1038/s41467-024-52108-9 PMC11377731

[24] K. S. Park , Z. Ni , A. P. Côté , J. Y. Choi , R. Huang , F. J. Uribe‐Romo , H. K. Chae , M. O'Keeffe , O. M. Yaghi , Proc. Natl. Acad. Sci. USA 2006, 103, 10186. 16798880 10.1073/pnas.0602439103 PMC1502432

[25] Y. Zhang , J. Guan , X. Wang , J. Yu , B. Ding , ACS Appl. Mater. Interfaces 2017, 9, 41087. 29087181 10.1021/acsami.7b14635

[26] Y.‐H. Huang , M.‐J. Wang , T.‐S. Chung , Nat. Commun. 2024, 15, 1092. 38316772 10.1038/s41467-024-45414-9 PMC10844271

[27] Y. H. Huang , S. Seenuvasan , M. J. Wang , T. S. Chung , Chem. Eng. J. 2025, 505, 159147.

[28] Z. Cui , N. T. Hassankiadeh , Y. Zhuang , E. Drioli , Y. M. Lee , Prog. Polym. Sci. 2015, 51, 94.

[29] H. Bux , A. Feldhoff , J. Cravillon , M. Wiebcke , Y.‐S. Li , J. Caro , Chem. Mater. 2011, 23, 2262.

[30] S. Turner , O. I. Lebedev , F. Schröder , D. Esken , R. A. Fischer , T. G. Van , Chem. Mater. 2008, 20, 5622.

[31] D. D. Kachhadiya , Z. V. P. Murthy , Environ Sci (Camb) 2023, 9, 1502.

[32] H. Sun , Z. Magnuson , W. He , W. Zhang , H. Vardhan , X. Han , G. He , S. Ma , ACS Appl. Mater. Interfaces 2020, 12, 20664. 32227857 10.1021/acsami.0c02513

[33] H. G. Yang , C. H. Sun , S. Z. Qiao , J. Zou , G. Liu , S. C. Smith , H. M. Cheng , G. Q. Lu , Nature 2008, 453, 638. 18509440 10.1038/nature06964

[34] X. Ma , R. J. Swaidan , Y. Wang , C. Hsiung , Y. Han , I. Pinnau , ACS Appl. Nano Mater. 2018, 1, 3541.

[35] A. A. Ismail , D. W. Bahnemann , I. Bannat , M. Wark , J. Phys. Chem. C 2009, 113, 7429.

[36] I. Vamvasakis , K. S. Subrahmanyam , M. G. Kanatzidis , G. S. Armatas , ACS Nano 2015, 9, 4419. 25871841 10.1021/acsnano.5b01014

[37] M. Thommes , K. Kaneko , A. V. Neimark , J. P. Olivier , F. Rodriguez‐Reinoso , J. Rouquerol , K. S. W. Sing , Pure Appl. Chem. 2015, 87, 1051.

[38] S. Cao , G. Gody , W. Zhao , S. Perrier , X. Peng , C. Ducati , D. Zhao , A. K. Cheetham , Chem. Sci. 2013, 4, 3573.

[39] M. E. Leitch , C. Li , O. Ikkala , M. S. Mauter , G. V. Lowry , Environ. Sci. Technol. Lett. 2016, 3, 85.

[40] T. He , R. Jia , X. Lang , X. Wu , Y. Wang , J. Electrochem. Soc. 2017, 164, E379.

[41] K. Zhang , R. P. Lively , C. Zhang , R. R. Chance , W. J. Koros , D. S. Sholl , S. Nair , J. Phys. Chem. Lett. 2013, 4, 3618.

[42] E. E. Sann , Y. Pan , Z. Gao , S. Zhan , F. Xia , Sep. Purif. Technol. 2018, 206, 186.

[43] D. Kim , D. W. Kim , O. Buyukcakir , M.‐K. Kim , K. Polychronopoulou , A. Coskun , Adv. Funct. Mater. 2017, 27, 1700706.

[44] K. Jayaramulu , K. K. R. Datta , C. Rösler , M. Petr , M. Otyepka , R. Zboril , R. A. Fischer , Angew. Chem., Int. Ed. 2016, 55, 1178. 10.1002/anie.201507692 26639893

[45] H. A. Hijaz , M. Zargar , A. Shafieian , A. Razmjou , M. Khiadani , J Memb Sci 2023, 687, 122089.

[46] H. A. Hijaz , M. Zargar , A. Shafieian , A. Razmjou , M. Khiadani , Sep. Purif. Technol. 2024, 350, 127978.

[47] W. Shang , H. Chen , G. Lu , X. Xu , M. Cheng , C. Huang , X. Guan , A. K. An , J Memb Sci 2025, 735, 124508.

[48] T. Horseman , Y. Yin , K. S. S. Christie , Z. Wang , T. Tong , S. Lin , ACS ES&T Engineering 2021, 1, 117.

[49] R. Hu , L. Hou , J. Liu , Z. Cui , B. Miao , J. Bai , X. Man , N.ü Wang , L. Jiang , Y. Zhao , J Mater Chem A Mater 2023, 11, 1704.

[50] W. Zhang , G. Liang , S. Wang , F. Yang , X. Liu , J. Yu , et al., Adv. Funct. Mater. 2024, 35, 2412424.

[51] B. Wicklein , A. Kocjan , G. Salazar‐Alvarez , F. Carosio , G. Camino , M. Antonietti , L. Bergström , Nat. Nanotechnol. 2015, 10, 277. 25362476 10.1038/nnano.2014.248

[52] Z.‐L. Yu , N. Yang , V. Apostolopoulou‐Kalkavoura , B. Qin , Z.‐Y. Ma , W.‐Y.i Xing , C. Qiao , L. Bergström , M. Antonietti , S.‐H. Yu , Angew. Chem., Int. Ed. 2018, 57, 4538. 10.1002/anie.201711717 29469238

[53] X. Meng , C. Liu , J. Zhang , W. Guo , N. Li , Y. Chen , H. Xu , M. Xi , S. Zhang , Z. Wang , J Mater Chem A Mater 2024, 12, 16079. 10.1021/acsami.4c08796 39036935

[54] J. Zhang , Y. Cheng , C. Xu , M. Gao , M. Zhu , L. Jiang , Adv. Funct. Mater. 2021, 31, 2009349.

[55] J. H. Park , S. Jang , Y. N. Kim , J. Choi , H. Yeo , K. H. Nam , Polym. Test. 2025, 147, 108797.

[56] H. Li , C. Zhuo , F. Zou , D. Wang , S. Xu , P. Xu , Z. Liu , F. Lv , X. Wang , C. Li , H. Feng , S. Zhang , X. Jian , Chem. Eng. J. 2024, 492, 152430.

[57] N. Mehra , L. Mu , T. Ji , X. Yang , J. Kong , J. Gu , J. Zhu , Appl. Mater. Today 2018, 12, 92.

[58] M. M. Mohseni , M. Jouyandeh , S. M. Sajadi , A. Hejna , S. Habibzadeh , A. Mohaddespour , N. Rabiee , H. Daneshgar , O. Akhavan , M. Asadnia , M. Rabiee , S. Ramakrishna , R. Luque , M. R. Saeb , Chem. Eng. J. 2022, 449, 137700.

[59] C. Mu , H. Chen , X. Sun , G. Liu , K. Yan , Prog. Org. Coat. 2021, 161, 106465.

[60] A. Karimi , A. Khataee , V. Vatanpour , M. Safarpour , Sep. Purif. Technol. 2019, 229, 115838.

[61] P. Cai , J. Li , D. Y. Song , N. Zhang , N. Wang , Q. F. An , J Memb Sci 2024, 695, 122489.

[62] A. Karimi , V. Vatanpour , A. Khataee , M. Safarpour , J. Ind. Eng. Chem. 2019, 73, 95.

[63] A. Sotto , G. Orcajo , J. M. Arsuaga , G. Calleja , J. Landaburu‐Aguirre , J. Appl. Polym. Sci. 2015, 132, 41633, 10.1002/app.41633.

[64] J. Zhou , S. Lin , H. Zeng , J.i Liu , B. Li , Y. Xu , X. Zhao , G. Chen , Mater. Horiz. 2020, 7, 2936.

[65] A. Anvari , A. A. Yancheshme , K. M. Kekre , A. Ronen , J Memb Sci 2020, 616, 118413.

[66] M. Qtaishat , T. Matsuura , B. Kruczek , M. Khayet , Desalination 2008, 219, 272.

[67] J.‐G. Lee , E.‐J. Lee , S. Jeong , J. Guo , A. K. An , H. Guo , J. Kim , T. Leiknes , N. Ghaffour , J Memb Sci 2017, 526, 395.

[68] G. S. Larsen , P. Lin , K. E. Hart , C. M. Colina , Macromolecules 2011, 44, 6944.

[69] S. Plimpton , J. Comput. Phys. 1995, 117, 1, 10.1006/jcph.1995.1039.

[70] H. J. C. Berendsen , J. P. M. Postma , W. F. van Gunsteren , A. DiNola , J. R. Haak , J. Chem. Phys. 1984, 81, 3684.

[71] B. Zheng , M. Sant , P. Demontis , G. B. Suffritti , J. Phys. Chem. C 2012, 116, 933.

[72] L. S. Dodda , I. C. d. Vaca , J. Tirado‐Rives , W. L. Jorgensen , Nucleic Acids Res. 2017, 45, W331. 28444340 10.1093/nar/gkx312 PMC5793816

[73] L. S. Dodda , J. Z. Vilseck , J. Tirado‐Rives , W. L. Jorgensen , J. Phys. Chem. B 2017, 121, 3864. 28224794 10.1021/acs.jpcb.7b00272 PMC5813481

[74] Y. Wu , H. L. Tepper , G. A. Voth , J. Chem. Phys. 2006, 124, 024503. 16422607 10.1063/1.2136877

