# Supplementary material for: Thermal Shielding and Vapor Transport Enhancement in MOF‐Enabled Membranes for Membrane Distillation
Source: Adv Sci (Weinh). 2025 Nov 16;13(6):e10323. doi: 10.1002/advs.202510323 (PMC12866747; doi:10.1002/advs.202510323)
Supplement: Supplementary file 1 — Supporting Information [file ADVS-13-e10323-s001.docx]

Supporting Information

**Thermal Shielding and Vapor Transport Enhancement in MOF-Enabled Membranes for Membrane Distillation**

Xiaolu Li^†^, Shengming Yin^†^, Jianhao Qian^†^, Shahid Ali Khan, Wentao Shang, Jiawei Sun, Muhammad Usman Farid*, Gang Lu, Guang Wang, Bhaskar Jyoti Deka, Jiaxin Guo, Yanguang Zhou, Sunwoo Kim, Junghwan Kim, and Alicia Kyoungjin An*

^†^These authors contributed equally to this work.

*Corresponding author. Email: alicia.kjan@ust.hk; mufarid@ust.hk

Xiaolu Li^†^, Shengming Yin^†^, Jianhao Qian^†^, Shahid Ali Khan, Jiawei Sun, Gang Lu, Muhammad Usman Farid*, Alicia Kyoungjin An*

School of Energy and Environment, City University of Hong Kong, 83 Tat Chee Avenue, Kowloon, Hong Kong SAR, China

**Muhammad Usman Farid*****, Alicia Kyoungjin An***

Department of Chemical and Biological Engineering, The Hong Kong University of Science and Technology, Clear Water Bay, Kowloon, Hong Kong SAR, China

**Jianhao Qian†**

Department of Civil and Environmental Engineering, Rice University, Houston, TX 77005, USA.

**Wentao Shang**

Energy and Electricity Research Center, International Energy College, Jinan University, Guangdong 519070, China

**Guang Wang, Yanguang Zhou**

Department of Mechanical and Aerospace Engineering, The Hong Kong University of Science and Technology, Clear Water Bay, Kowloon, Hong Kong SAR, China

**Shengming Yin^†^**

State Key Laboratory of Materials Processing and Die & Mould Technology, Department of Materials Science and Engineering, Huazhong University of Science and Technology, Wuhan, Hubei 430074, China

**Bhaskar Jyoti Deka**

Department of Hydrology, Indian Institute of Technology Roorkee, Haridwar, Uttarakhand, 247667, India

Jiaxin Guo

School of Chemical Engineering and Technology, Xi’an Jiaotong University, Xi’an, 710049, China

Sunwoo Kim, Junghwan Kim

Department of Chemical and Biomolecular Engineering, Yonsei University, 50 Yonsei-ro, Seodaemun-gu, Seoul, 03722, South Korea

**This PDF file includes:**

Supplementary Text

Figures S1 to S15

Tables S1 to S3

References

Supplementary Text

**Molecular dynamics simulations**

Molecular dynamics simulations were employed to analyze the flux, water transport mechanisms, and temperature profile in nanoscale channels constructed with PH or ZIF-8 walls. The ZIF-8 walls were constructed by extending the structure to include two unit cells along the X-axis (approximately 3.4 nm), one unit cell along the Y-axis (approximately 2.6 nm), and five unit cells along the Z-axis (approximately 10 nm). For the PH polymer walls, 12 chains consisting of 20 repeating units of -[CH₂-CF₂]-[CF₂-CF(CH₃)]- were initially constructed. These were subjected to a 21-step molecular dynamic annealing process [1] resulting in walls with a thickness of 1.7 nm (along the Y-axis), a length of approximately 10 nm (along the Z-axis), and a periodic width of 3.4 nm (along the X-axis).

After constructing both ZIF-8 and PH walls, a 2 nm vacuum layer was created above the walls in the Y direction to form the nanoscale channels, resulting in ZIF-8 and PH channels with a height of 2 nm. To maintain structural integrity during the simulation, 10% of the atoms in the ZIF-8 and PH walls were tethered to their initial positions by applying spring forces. Once the nanoscale channels were created, 3 nm-thick water layers were placed on either side of the channels, and pistons applied 1 bar of pressure to these water layers to mimic real-world conditions. The water temperatures on both sides were controlled at 60°C and 20°C, respectively, while the overall system temperature was maintained at 40°C.

Under the temperature difference, the number of water molecules decreases on the hot side and increases on the cold side at a rate that becomes equal at steady state. After relaxing the entire system for 10 ns, the simulation was continued for an additional 60 ns to monitor the increase in the number of water molecules in the cold-side water layer. Flux was determined by calculating the rate of change in the number of water molecules over time. The simulation domain was divided into slices along the Z-axis with a thickness of 0.5 nm. The average temperature within each slice over the 60 ns of the steady-state simulation was calculated for the temperature profile.

All temperature controls were regulated using a Berendsen thermostat [2]. Atomic trajectories were calculated using the velocity-Verlet integrator with a 1 fs timestep. All simulations were conducted in LAMMPS [3]. The force field for ZIF-8 was obtained from literature [4,5]. The OPLS-AA force field was used for PH, with parameters assigned via the LigParGen server [6,7]. The SPC/E model was employed for water molecules [8].

**CFD analysis**

The commercial software ANSYS Fluent was used to simulate the MD system. A structured quadrilateral mesh with element sizes ranging from 0.02 mm to 0.06 mm was utilized, with a total of 11,000 elements optimized for no significant temperature variation and a target skewness of 0.9. The simulations were conducted under non-isothermal conditions. The feed inlet was set at 60 °C, and the permeate inlet was at 20 °C. Ambient atmospheric pressure was maintained at the channel outlets. No-slip velocity conditions were enforced at all wall surfaces, with thermal coupling across the membrane walls facilitated by a user-defined function (UDF) that applied heat flux on both the feed and permeate sides of the membrane walls. The Navier-Stokes and energy equations were solved using the SIMPLE scheme for pressure-velocity coupling and the second-order upwind technique for the continuity, momentum, and energy equations. This approach ensures high accuracy in flow and thermal fields. The convergence criteria for the normalized residuals of continuity, velocities, and energy were stringently set below 10^-6^, minimizing linearization errors and enhancing simulation robustness. A laminar fluid flow model was employed in the analysis, as the maximum Reynolds number of 1592 indicates a laminar flow regime.


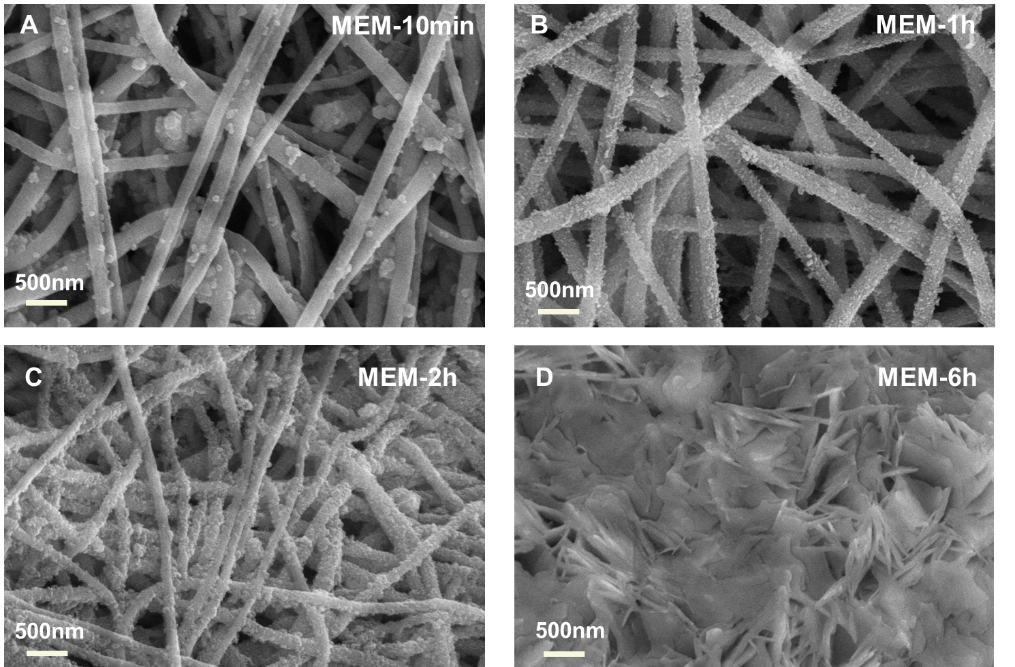


**Figure S1.** Loading of ZIF-8 at different growth times: (A) 10 min, (B) 1 hour, (C) 2 hours, and (D) 6 hours.


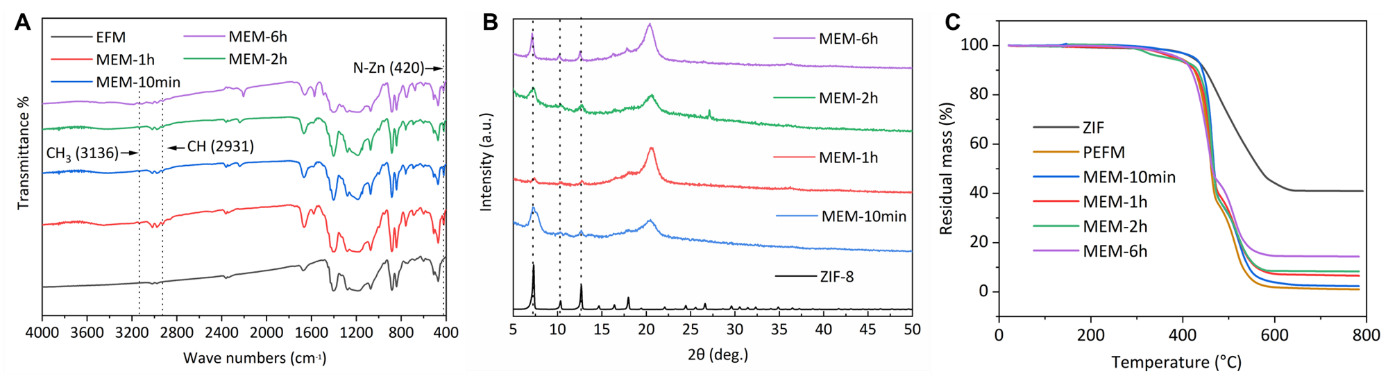


Figure S2. (A) FTIR spectra of the MEMs and PH/PVP EFM. Four MEM membranes exhibit the characteristic ZIF-8 peaks corresponding to N–Zn (~420 cm⁻¹), CH₃ (~3136 cm⁻¹), and C–H (~2931 cm⁻¹), whereas the corresponding peaks are absent in the EFM. (B) XRD patterns of ZIF-8 powder and the MEMs. The diffraction peaks of ZIF-8 can be observed in the XRD pattern of the MEMs. (C) TGA analysis of ZIF-8, PEFM, and MEMs. The ZIF-8 loading on EFM gradually increased from 10 minutes to 6 hours, as reflected by the residual mass.


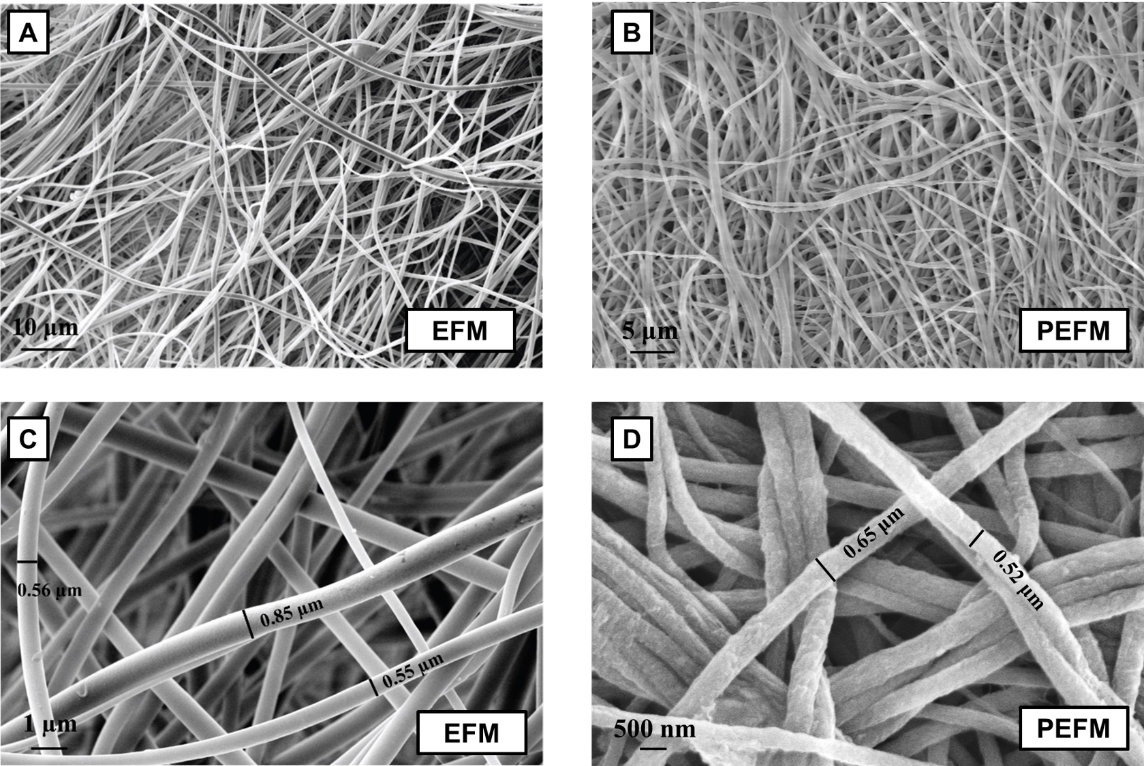


Figure S3. SEM images of (A) EFM and (B) PEFM at low magnification. An interconnected network of polymer fibers can be clearly observed in both samples. (C) EFM and (D) PEFM at high magnification. Dense polymer fibers are shown in EFM with three typical fibers with diameter of 0.56 µm, 0.79 µm and 0.55 µm. Mesoporous fibers are observed in the PEFM, with similar sizes of 0.65µm and 0.52 µm.


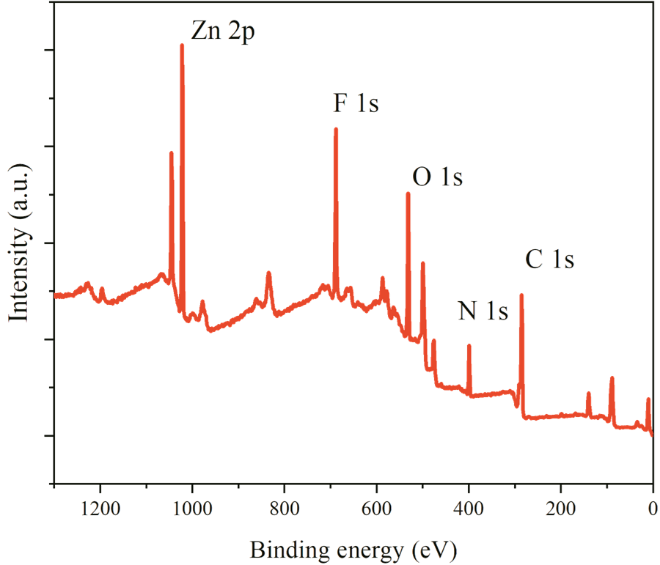


Figure S4. XPS survey spectrum of the MEM. C, N, O, F, and Zn are the main elements found in the MEM.


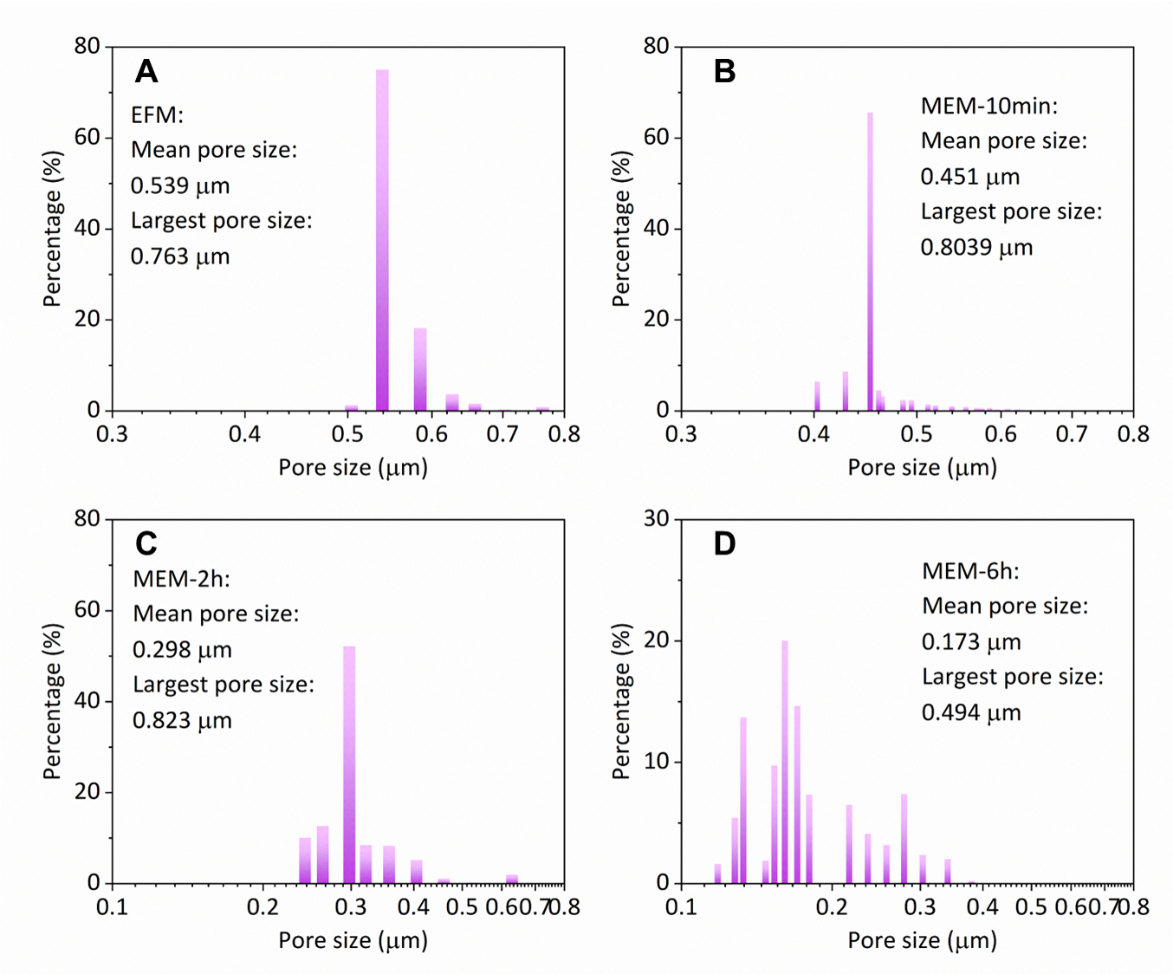


Figure S5. PSD of (A) EFM (B) MEM-10min (C) MEM-2h (D) MEM-6h by liquid-gas replacement method. The mean pore size of the EFM is approximately 0.54 μm, which is inherited by the MEM. The mean pore size of the MEM decreased as the growth time increased, attributed to the ZIF-8 layer deposited on the nanofibers, which increased the fiber diameter and consequently reduced the inter-fiber spacing. Furthermore, excessive ZIF-8 growth led to the agglomeration of some fibers while displacing others, thereby decreasing the mean pore size while broadening the pore size distribution.


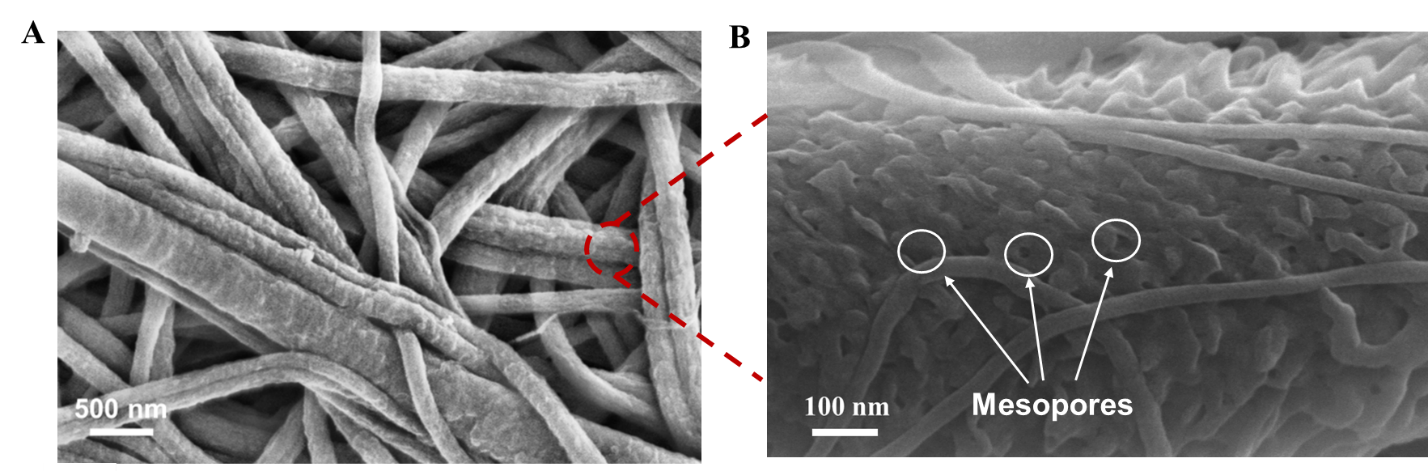


Figure S6. SEM images of the (A) fibrous network and (B) porous PH fiber of the PEFM. In (A), the rough, porous nanofiber-stacked micropores can be clearly seen, and the mesopores left on the nanofibers due to PVP removal can be clearly observed in (B).





Figure S7. Tensile strength test results of the MEM, PEFM, and C-PVDF. Due to the difference in fabrication methods, the porosity of the C-PVDF is lower, which consequently results in a higher mechanical strength. The nanofiber structures of the PEFM and MEM are relatively loose, leading to a lower mechanical strength.


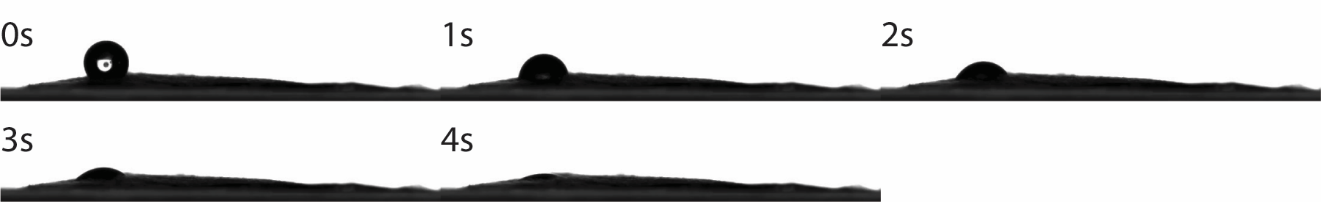


Figure S8. Water contact angle of the EFM. Water droplet is fully adsorbed by the EFM after 4 seconds.


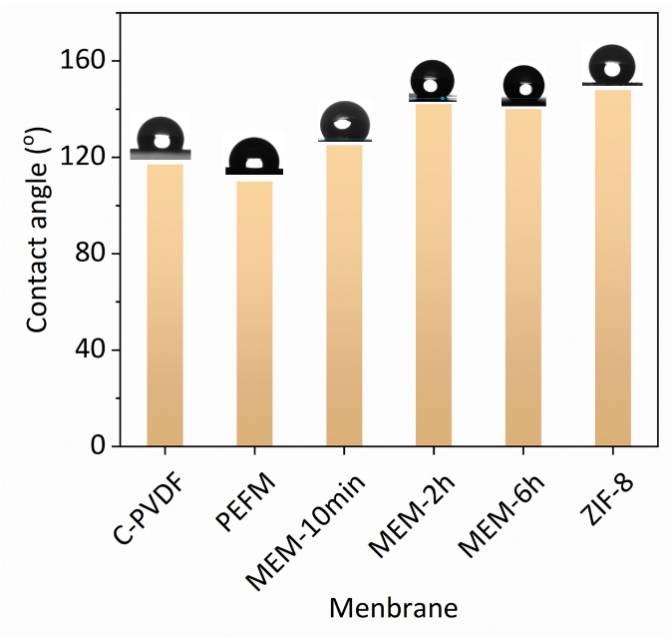


Figure S9. Water contact angles of the C-PVDF, PEFM, MEMs and ZIF-8 powder.


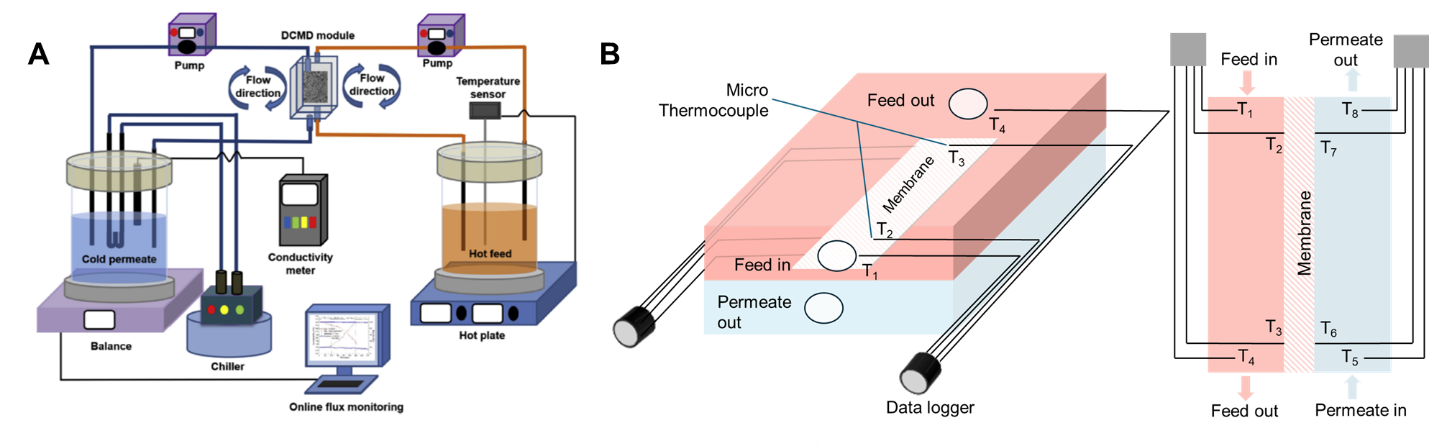


Figure S10. Schematic of (A) customized DCMD set-up, (B) membrane module with temperature monitoring. The feed solution is heated using a hot plate, and permeate water is cooled by a chiller. The feed and permeate are circulated in a opposite direction across the membrane. The flux is monitored by the weight change of permeate tank using an electric balance. The salt rejection is calculated by the conductivity change of permeate. The temperature profile is monitored by eight thermocouple sensors. Real-time temperature data were collected at eight monitoring points (T1 to T8): feed inlet bulk (T1), feed inlet membrane surface (T2), feed outlet membrane surface (T3), feed outlet bulk (T4), permeate inlet bulk (T5), permeate inlet membrane surface (T6), permeate outlet membrane surface (T7), and permeate outlet (T8).

**
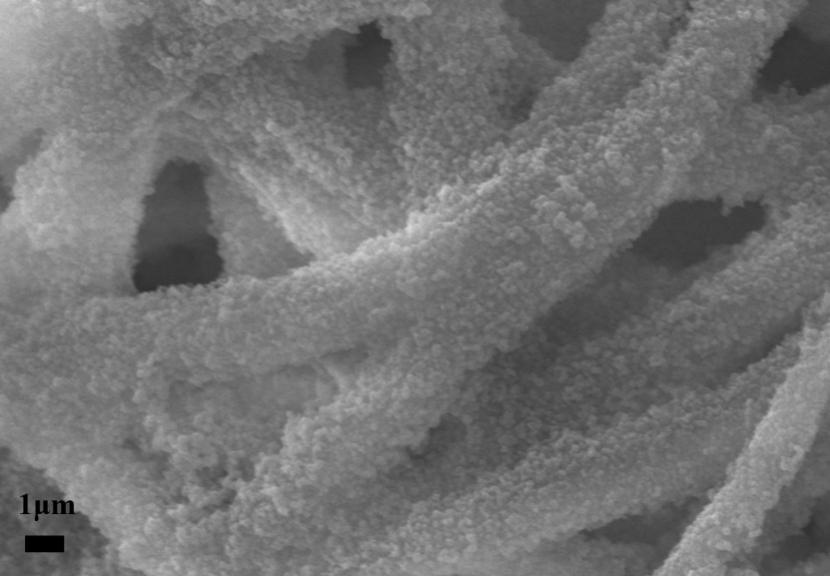
**

**Figure S11. SEM image of the MEM after durability test.** The complete ZIF-8 nanocrystal layer remained after long-term test, indicating that the flowing hot saline water causing no significant damage to the MEM’s surface structure.


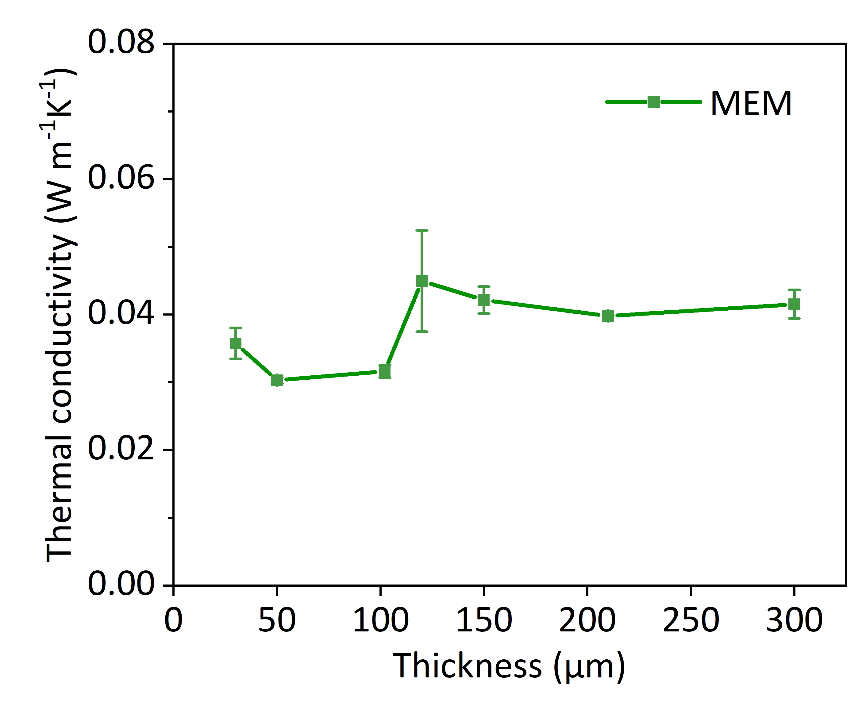


**Figure S12. Thermal conductivity measurements of MEM membranes with different thicknesses.**

*
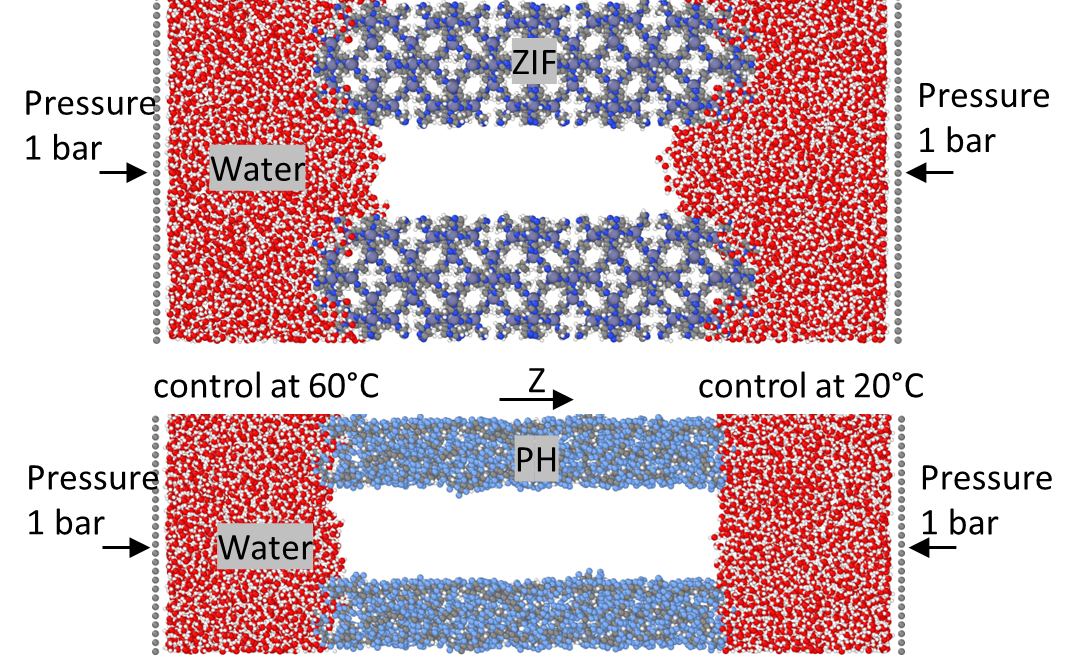
*

**Figure S13. Molecular dynamics simulations of ZIF-8 and PH channels.** The red areas on both sides represent gaseous water molecules, while the walls of the channel are composed of porous ZIF-8 and chain-like PH molecules. By controlling the temperature and pressure of the water molecules at both ends, the behavior of the water molecules diffusing from the high-temperature region to the low-temperature region can be observed.

**
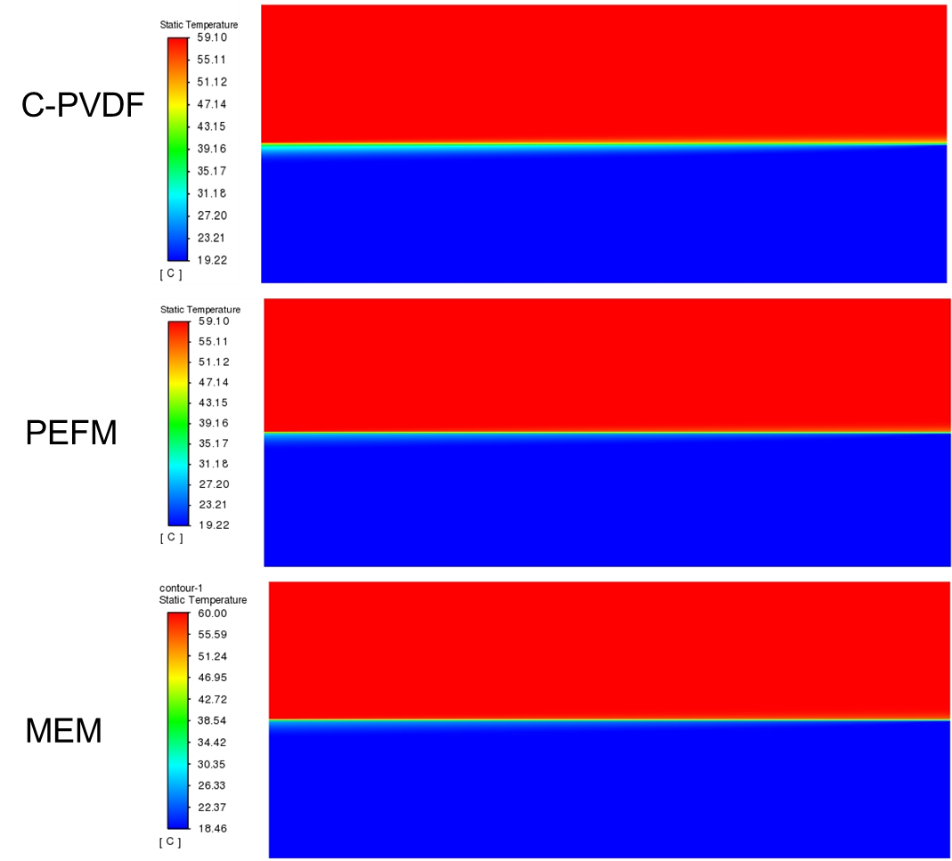
**

**Figure S14. Temperature contours generated by CFD.** CFD was used to simulate the surface temperature distribution of three membranes along the flow direction, with a feed temperature of 60°C and a permeate temperature of 20°C. The red area represents the feed bulk, the blue area represents the permeate bulk, and the transition zone in between is the membrane region. It can be observed in the temperature profiles that the stagnant layer along the C-PVDF is thicker than in the MEM, indicating a more severe temperature polarization.

**
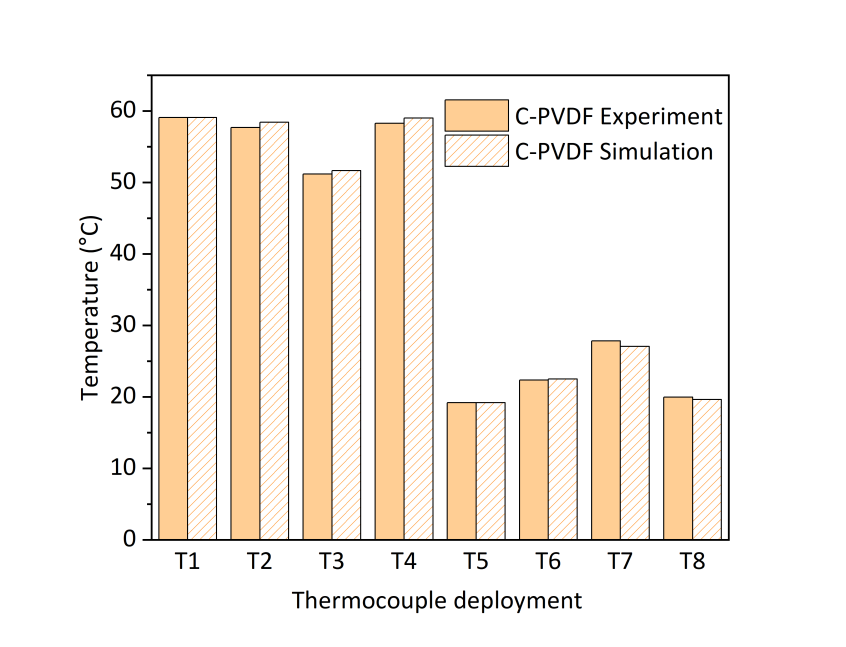
**

**Figure S15. Comparison of experimental and CFD simulated temperatures for the C-PVDF at 60 ℃.** The temperatures at each control point obtained from CFD simulations are consistent with the experimental measurements, validating the accuracy of the model.

Table S1. Measured temperature, flux, and thermal efficiency during MD operation

|  | **T1 (℃)** | **T2 (℃)** | **T3 (℃)** | **T4 (℃)** | **T5 (℃)** | **T6 (℃)** | **T7 (℃)** | **T8 (℃)** | **Flux (LMH)** | **Thermal efficiency (%)** | |
| --- | --- | --- | --- | --- | --- | --- | --- | --- | --- | --- | --- |
| C-PVDF | 59.10 | 57.70 | 51.19 | 58.30 | 19.22 | 22.35 | 27.84 | 19.97 | 18.7 | | 35.7 |
| PEFM | 58.97 | 58.00 | 52.12 | 58.38 | 19.97 | 21.56 | 22.17 | 20.76 | 32.7 | | 54.1 |
| MEM-10min | 59.12 | 58.32 | 52.99 | 58.51 | 19.96 | 21.64 | 22.23 | 20.63 | 35.3 | | 63.8 |
| MEM-1h | 59.54 | 58.55 | 57.79 | 58.49 | 19.63 | 21.27 | 22.34 | 21.16 | 44.5 | | 71.3 |
| MEM-2h | 60.16 | 58.79 | 57.98 | 58.97 | 19.93 | 21.67 | 23.18 | 23.22 | 40.3 | | 67.9 |
| MEM-6h | 55.36 | 57.71 | 53.13 | 55.72 | 19.78 | 22.39 | 21.85 | 19.89 | 12.1 | | 29.5 |

Table S2. Characteristics comparison of MEM and other membranes

| **Membrane name** | **Mean pore size (µm)** | **Thickness (µm)** | **Porosity (%)** | **Contact angle (°)** | **LEP (kPa)** | **Thermal conductivity (W m^-1^ K^-1^)** | **Water flux (LMH)** | **Thermal efficiency (%)** | **Ref** |
| --- | --- | --- | --- | --- | --- | --- | --- | --- | --- |
| **ECTFE, 3M™** | 0.43 | 46 | 67 | 118 | n.a. | 0.034 | 42 | 60 | [9] |
| **0.45 PP, 3M™** | 0.79 | 110 | 5 | 130 | n.a. | 0.048 | 40 | 58 | [9] |
| **2400, Celgard™** | 0.043 | 25 | 41 | 138 | n.a. | 0.111 | 3.9 | 3 | [9] |
| **0.22PP, Tisch** | 1.79 | 196 | 72 | 119 | 42.7 | 0.066 | 24 | 44 | [10] |
| **0.45PP, Tisch** | 2.65 | 175 | 72 | 125 | 38.6 | 0.066 | 24 | 39 | [10] |
| **0.22PTFE, Tisch** | 0.33 | 188 | 75 | 121 | 126 | 0.082 | 42 | 53 | [10] |
| **0.45PTFE, Tisch** | 0.36 | 156 | 78 | 117 | 133 | 0.075 | 55 | 59 | [10] |
| **Nanocellulose** | 0.28 | 502 | 89 | 144 | 74.7 | 0.040 | 23 | 71 | [10] |
| **PVDF-0.22, Millipore™ GVHP** | 0.22 | 125 | 50 | 118 | 246 | 0.0688 | 20.6 | 39.54 | This work |
| **PVDF-0.45, Millipore™ HVHP** | 0.45 | 120 | 60 | 120 | 135 | 0.0710 | 18.7 | 35.69 | This work |
| **PEFM** | 0.56 | 105 | 85 | 110 | 145 | 0.0481 | 32.7 | 54.01 | This work |
| **MEM-10min** | 0.45 | 110 | 83 | 125 | 150 | 0.0436 | 35.3 | 63.89 | This work |
| **MEM-1h** | 0.47 | 102 | 90 | 147 | 157 | 0.0316 | 44.5 | 71.30 | This work |
| **MEM-2h** | 0.29 | 109 | 85 | 142 | 162 | 0.0386 | 40.3 | 67.95 | This work |
| **MEM-6h** | 0.17 | 98 | 65 | 140 | 191 | 0.0567 | 12.1 | 29.58 | This work |

Table S3. Comparison of long-term performance of with membranes reported in the literature.

| **Membrane** | **Salt Conc**  **(wt%)** | **Temperature**  **difference（℃）** | **Rejection**  **(%)** | **Flux**  **(LMH)** | **Long-term**  **Durability (h)** | **Ref** |
| --- | --- | --- | --- | --- | --- | --- |
| **Nanocellulose** | 1 | 40 | 99.8 | 23 | 8 | [10] |
| **PVDF/MAF-4** | 3.5 | 40 | 99.9 | 27.9 | 6 | [11] |
| **PVDF-chitosan/ZIF-8** | 3.5 | 40 | 99.9 | 7.2 | 15 | [12] |
| **PH/AlFu MOF** | 3.5 | 40 | 99.9 | 22.7 | 48 | [13] |
| **PVDF/MOF-F300** | 3.5 | 32 | 99.99 | 2.87 | 5 | [14] |
| **PVDF/PTFE** | 3.5 | 65 | 99.99 | 35.6 | 20 | [15] |
| **PVDF/PTFE** | 3.5 | 40 | 99.90 | 29.1 | 15 | [16] |
| **PVDF-0.22, Millipore™ GVHP** | 3.5 | 40 | 99.99 | 20.6 | 32 | This work |
| **PVDF-0.45, Millipore™ HVHP** | 3.5 | 40 | 99.99 | 18.7 | 30 | This work |
| **PEFM** | 3.5 | 40 | 99.99 | 32.7 | 35 | This work |
| **MEM** | 3.5 | 40 | 99.99 | 44.5 | 40 | This work |

Reference

[1] Larsen GS, Lin P, Hart KE, Colina CM. Molecular Simulations of PIM-1-like Polymers of Intrinsic Microporosity. Macromolecules 2011;44:6944–51. https://doi.org/10.1021/ma200345v.

[2] Berendsen HJC, Postma JPM, van Gunsteren WF, DiNola A, Haak JR. Molecular dynamics with coupling to an external bath. J Chem Phys 1984;81:3684–90. https://doi.org/10.1063/1.448118.

[3] Plimpton S. Fast Parallel Algorithms for Short-Range Molecular Dynamics. J Comput Phys 1995;117:1–19. https://doi.org/https://doi.org/10.1006/jcph.1995.1039.

[4] Zheng B, Sant M, Demontis P, Suffritti GB. Force Field for Molecular Dynamics Computations in Flexible ZIF-8 Framework. The Journal of Physical Chemistry C 2012;116:933–8. https://doi.org/10.1021/jp209463a.

[5] Park KS, Ni Z, Côté AP, Choi JY, Huang R, Uribe-Romo FJ, et al. Exceptional chemical and thermal stability of zeolitic imidazolate frameworks. Proceedings of the National Academy of Sciences 2006;103:10186–91. https://doi.org/10.1073/pnas.0602439103.

[6] Dodda LS, Cabeza de Vaca I, Tirado-Rives J, Jorgensen WL. LigParGen web server: an automatic OPLS-AA parameter generator for organic ligands. Nucleic Acids Res 2017;45:W331–6. https://doi.org/10.1093/nar/gkx312.

[7] Dodda LS, Vilseck JZ, Tirado-Rives J, Jorgensen WL. 1.14*CM1A-LBCC: Localized Bond-Charge Corrected CM1A Charges for Condensed-Phase Simulations. J Phys Chem B 2017;121:3864–70. https://doi.org/10.1021/acs.jpcb.7b00272.

[8] Wu Y, Tepper HL, Voth GA. Flexible simple point-charge water model with improved liquid-state properties. J Chem Phys 2006;124:024503. https://doi.org/10.1063/1.2136877.

[9] Vanneste J, Bush JA, Hickenbottom KL, Marks CA, Jassby D, Turchi CS, et al. Novel thermal efficiency-based model for determination of thermal conductivity of membrane distillation membranes. J Memb Sci 2018;548:298–308. https://doi.org/10.1016/J.MEMSCI.2017.11.028.

[10] Hou D, Li T, Chen X, He S, Dai J, Mofid SA, et al. Hydrophobic nanostructured wood membrane for thermally efficient distillation. Sci Adv 2019;5:eaaw3203. https://doi.org/10.1126/sciadv.aaw3203.

[11] Wu R, Tan Y, Meng F, Zhang Y, Huang YX. PVDF/MAF-4 composite membrane for high flux and scaling-resistant membrane distillation. Desalination 2022;540:116013. https://doi.org/10.1016/J.DESAL.2022.116013.

[12] Kebria MRS, Rahimpour A, Bakeri G, Abedini R. Experimental and theoretical investigation of thin ZIF-8/chitosan coated layer on air gap membrane distillation performance of PVDF membrane. Desalination 2019;450:21–32. https://doi.org/10.1016/J.DESAL.2018.10.023.

[13] Wu XQ, Mirza NR, Huang Z, Zhang J, Zheng YM, Xiang J, et al. Enhanced desalination performance of aluminium fumarate MOF-incorporated electrospun nanofiber membrane with bead-on-string structure for membrane distillation. Desalination 2021;520:115338. https://doi.org/10.1016/J.DESAL.2021.115338.

[14] Yang F, Efome JE, Rana D, Matsuura T, Lan C. Metal–Organic Frameworks Supported on Nanofiber for Desalination by Direct Contact Membrane Distillation. ACS Appl Mater Interfaces 2018;10:11251–60. https://doi.org/10.1021/acsami.8b01371.

[15] Pan J, Zhang F, Wang Z, Sun SP, Cui Z, Jin W, et al. Enhanced anti-wetting and anti-fouling properties of composite PFPE/PVDF membrane in vacuum membrane distillation. Sep Purif Technol 2022;282:120084. https://doi.org/10.1016/J.SEPPUR.2021.120084.

[16] Dong ZQ, Ma X hua, Xu ZL, You WT, Li F bing. Superhydrophobic PVDF–PTFE electrospun nanofibrous membranes for desalination by vacuum membrane distillation. Desalination 2014;347:175–83. https://doi.org/10.1016/J.DESAL.2014.05.015.
